# Impact of different formulations of platelet lysate on proliferative and immune profile of equine mesenchymal stromal cells

**DOI:** 10.3389/fvets.2024.1410855

**Published:** 2024-08-05

**Authors:** Kevin Yaneselli, Gimena Ávila, Andrea Rossi, Analía Rial, Sabrina Castro, María José Estradé, Gonzalo Suárez, Agustina Algorta

**Affiliations:** ^1^Unidad de Inmunología e Inmunoterapia, Departamento de Patobiología, Facultad de Veterinaria, Universidad de la República, Montevideo, Uruguay; ^2^Departamento de Desarrollo Biotecnológico, Facultad de Medicina, Universidad de la República, Montevideo, Uruguay; ^3^Unidad de Clínica y Cirugía de Equinos, Departamento de Clínica y Hospital Veterinario, Facultad de Veterinaria, Universidad de la República, Montevideo, Uruguay; ^4^Unidad de Reproducción Animal, Departamento de Producción Animal y Salud de los Sistemas Productivos, Facultad de Veterinaria, Universidad de la República, Montevideo, Uruguay; ^5^Unidad de Farmacología y Terapéutica, Departamento de Clínicas y Hospital Veterinario, Facultad de Veterinaria, Universidad de la República, Montevideo, Uruguay

**Keywords:** platelet lysate, horse, mesenchymal stromal/stem cells, fetal bovine serum, xeno-free media, immunology

## Abstract

Platelet lysate (PL) is investigated as a potential replacement for fetal bovine serum (FBS) in cell culture. However, there is limited research on its impact on the immune profile of equine mesenchymal stromal cells (eMSCs). This study aimed to evaluate the effects of different PL formulations on the proliferative capacity, multipotentiality, and immune profile of equine adipose tissue-derived MSCs (eAD-MSCs). *In vitro* growth kinetics and trilineage differentiation of eAD-MSCs (*n* = 7) were assessed under three culture conditions: medium-concentration PL (MPL), high-concentration PL (HPL), and FBS as a control. The immune profile was evaluated by studying the expression of immunogenic receptors such as MHC I, MHC II, and immunomodulatory molecules IL-6, IL-10, and TNF-α, determined by gene expression, surface marker expression, and cytokine quantification. Both PL formulations, pooled from 5 donors, exhibited 3.3 and 6.5-fold higher platelet counts than baseline plasma for MPL and HPL, respectively. Higher concentrations of TGF-β and PDGF were found in both PL formulations compared to baseline. Furthermore, MPL and HPL subcultures demonstrated proliferative, clonogenic, and multipotent capacities similar to FBS. The immune profile of PL-cultured cells exhibited gene expression levels related to immunogenicity and immunomodulation similar to the reference condition, and the surface antigen presence of MHC II was also similar. However, HPL media exhibited higher IL-6, IL-10, and TNF-α concentrations in the culture supernatant. In conclusion, both PL media contained higher concentrations of growth factors compared to FBS, supporting the *in vitro* culture of eAD-MSCs with proliferative, clonogenic, and multipotent capacity similar to the reference medium. Nonetheless, PL usage led to a variation in the immunomodulatory cytokine microenvironment, with higher concentrations of IL-6, IL-10, and TNF-α in HPL media compared to MPL and FBS.

## 1 Introduction

Mesenchymal stromal/stem cells (MSCs) have gained significant interest in veterinary medicine due to their ability to contribute to the structural and functional regeneration of injured tissues and their immunomodulatory effect that contributes to this therapeutic effect. These characteristics have led to different studies to prove their safety and therapeutic efficacy in different pathologies both in humans and in domestic animals (1). In the case of equines, there are precedents for the therapeutic application of MSCs, primarily in musculoskeletal pathologies as well as in other pathologies such as respiratory, reproductive, and ophthalmologic (2). In addition, their immunomodulatory effect has been proven *in vitro* (3) and *in vivo* (4), especially in some trials that explore the immunosuppressive effect in osteoarthritis in equines, finding an improvement in the joints treated with MSCs (5).

Before the therapeutic application of MSCs, *in vitro* isolation and propagation and the use of a culture medium are necessary. Fetal bovine serum (FBS) is used to stimulate cell growth, which is considered the “gold standard supplement” for cell cultures of animal origin (6). Relying on bovine-derived supplements for cells from different species raises safety concerns regarding therapy, as it increases the risk of an immunological xeno-reaction in patients receiving the cell therapy (6). Xeno-contamination of MSCs, caused by the inclusion of bovine proteins into their membranes, has been described. This leads to an immune response in recipients of cell therapy, as evidenced by detecting anti-FBS antibodies in both humans and domestic animals (7). Another drawback of utilizing FBS as a culture supplement is its impact on animal welfare. Obtaining FBS necessitates the sacrifice of bovine fetuses, and the extraction process entails their suffering (8).

In light of the therapeutic safety concerns and ethical controversies associated with the use of FBS, several studies have been conducted exploring replacements, one of them being platelet lysate (PL), which allows for the establishment of the so-called xeno-free culture, avoiding the xeno-contamination and ethical problems of the classic supplement (6, 9). Currently, the use of PL as a culture supplement for equine MSCs (eMSCs) has gained relevance in the literature, with recent studies exploring its feasibility for *in vitro* isolation and propagation, finding comparable results with cultures using FBS (10, 11). Moreover, it has been described that PL contains a high concentration of growth factors (GFs) such as transforming growth factor-β (TGF-β) and platelet-derived growth factors (PDGF), which are relevant in cell proliferation (10, 12). Nevertheless, high amounts of pro-inflammatory cytokines such as interleukin 1 beta (IL-1β) and tumor necrosis factor-alpha (TNF-α) have also been found, which may stimulate MSCs, promoting their immunomodulatory profile. The presence of immunomodulatory cytokines such as IL-6 and IL-10 has also been described (11, 13, 14).

However, there is currently no consensus or standardized approach regarding the method of platelet extraction, platelet concentration, and the proportion of PL in the culture medium. The existing literature shows significant heterogeneity in these aspects (7, 10). One of the most employed methods to obtain PL in equines is the double centrifugation technique (11, 15), renowned for its simplicity and minimal equipment requirement. Conversely, more sophisticated approaches exist, such as platelet extraction by plateletpheresis, which enables precise platelet separation and mitigates contamination with leukocytes (16, 17). Furthermore, there is variation regarding the optimal platelet concentration for PL production as a culture supplement. While some studies advocate for a reference concentration of 1 × 10^6^ platelets/μL (11, 12, 18), others have demonstrated comparable results with lower concentrations, achieving enhanced production efficiency (10, 16, 19, 20).

Few studies evaluate the impact of using PL as a supplement on the multipotentiality and immunomodulation of eMSCs, which are relevant characteristics for their therapeutic application. The immunogenicity and immunomodulatory profile of eMSC cultured with PL has been studied recently with conflicting results. In some studies, these properties were similar to cells cultured with classical supplementation (11, 16), while in others, alterations in the immune profile have been described, such as higher concentrations of IL-10 and TNF-α (14). Results from our group also describe an increased expression of IL-6 at the gene level when cultured with PL (19). The present study aims to investigate the impact of different formulations of allogeneic PL on the proliferative capacity, multipotentiality, and immune profile of equine adipose tissue-derived MSCs (eAD-MSCs). We hypothesize that PL can be used as a culture supplement for eMSCs at different high and medium platelet concentrations, yielding similar results among the different concentrations and comparable to FBS. Additionally, the profile of the eMSCs will be modified due to the incorporation of PL into the culture, which introduces a higher concentration of pro-inflammatory cytokines into the culture medium. This alteration could affect both the immunogenic and immunomodulatory profiles of the cells. Finally, the subculture of eMSCs with PL will not affect the proliferative capacity or the immune profile across passages.

## 2 Materials and methods

### 2.1 Preparation of allogeneic PL

**Figure 1:**
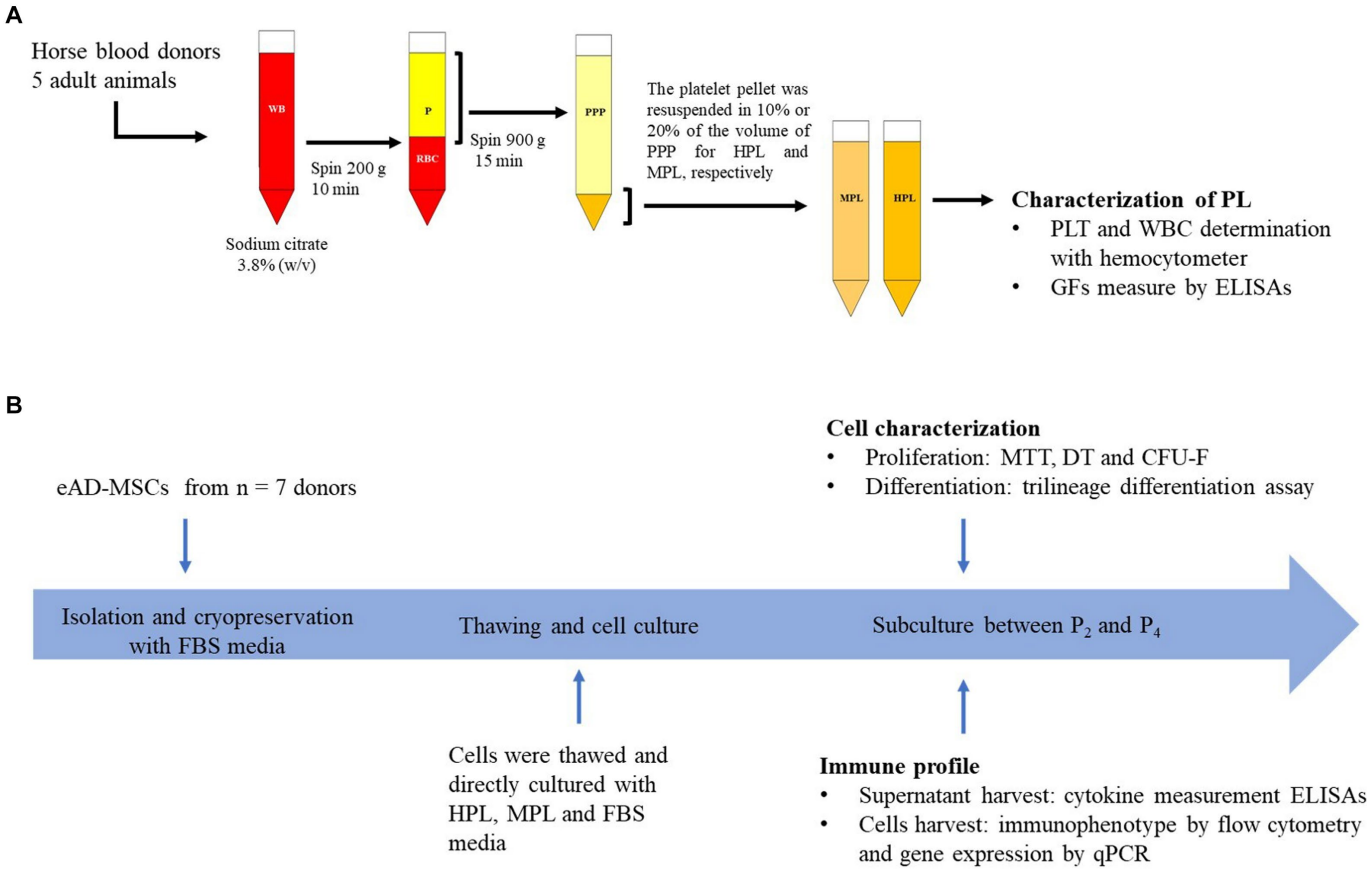
Overview of study design. **(A)** Preparation of allogeneic platelet lysate (PL). Once the plasma was collected, it was divided to formulate medium-concentration PL (MPL) and high-concentration PL (HPL). **(B)** Design and timeline of the cell culture experiment for equine adipose tissue-derived MSCs (eAD-MSCs). WB, whole blood; P, plasma; RBC, red blood cells; PPP, platelet-poor plasma; PLT, platelet; WBC, white blood cell; GFs, growth factors; FBS, fetal bovine serum.

The PL preparation protocol was adapted to those previously described in the literature (15, 19). Briefly, for the preparation of the PL, 500 mL of whole blood (WB) was extracted from the jugular vein in an aseptic manner from adult equines (*n* = 5), 4 males and 1 female, crossbred, healthy, with an age range of 3–5 years, the anticoagulant used was sodium citrate 3.8% (w/v). The samples were centrifuged at 200 *g* for 10 min, and the supernatant (plasma) was removed without lifting part of the leukocyte layer. This plasma fraction was subjected to a second centrifugation at 900 *g* for 15 min. Once the platelet pellet was obtained, two platelets concentrate (PC) formulations were prepared by resuspending them with the platelet-poor plasma resulting from the second centrifugation. For the medium-concentration PL (MPL) supplement, the pellet was resuspended in 20% of the initial total plasma volume until a concentration of 5× was reached. For the high-concentration PL (HPL) supplement, the pellet was resuspended in 10% of the initial total plasma volume to achieve a concentration of 10×. The platelet and leukocyte count of the basal plasma sample and the PC were determined using a hemocytometer (Mythic 18 Vet, Orphee). To lyse the platelets, a cycle of freezing at −80°C and thawing in a water bath at 37°C for 30 min was performed, followed by centrifugation at 1,600 *g* for 30 min. Finally, the different formulations obtained were filtered through 0.22 μm, and a PL pool was formed for each formulation. The study design is displayed in Figure 1A.

### 2.2 Quantification of PL growth factors

The concentrations of GFs, including TGF-β and PDGF-BB, in plasma, PC formulations (MPC and HPC), and PL formulations (MPL and HPL) were quantified in duplicate for each donor using Quantikine ELISA kits (DB100B and DBB00, R&D Systems). In addition, analysis of standard condition FBS (Capricorn) and Dulbecco’s modified Eagle’s medium (DMEM) (Gibco) used in culture medium was performed for comparison. Samples were stored at −80°C until quantification by ELISA was performed. The samples used for TGF-β were previously activated with hydrochloric acid, as suggested in the kit protocol. The samples were diluted 1:4 (TGF-β) and 1:20 (PDGF-BB) before starting the assay. The procedure performed for the quantification of both GFs followed the manufacturer’s recommendations, and the reading of the plates was performed through spectrophotometry at an optical density of 450 nm (Multiskan EX, Thermo Scientific).

### 2.3 Isolation and culture of eAD-MSCs

The first stage involved the isolation of eAD-MSCs. For this purpose, subcutaneous adipose tissue samples were taken from the abdominal region of healthy adult equines (*n* = 7), females, crossbred, weight 320–380 kg, age 3–6 years. The adipose tissue was collected during experimental surgery non-related to this study, using forceps and sterile surgical scissors, taking between 5 and 10 grams and transported under refrigeration (4–8°C) in a sterile tube with transport medium (DMEM + 20% (v/v) FBS) and processed in a maximum time of 24 h. Isolation consisted of washing twice with phosphate-buffered saline (PBS) supplemented with 2% (v/v) penicillin/amphotericin B (Capricorn) prior to mincing the tissue into 1 mm portions in a Petri dish. Subsequently, enzymatic digestion was performed for 40 min at 37°C using 0.1 mg/mL type I collagenase (Gibco) resuspended in low glucose DMEM. The digestion was stopped with culture medium supplemented with 20% FBS and centrifuged at 700 *g* for 10 min. The resulting pellet was seeded in culture medium flasks with DMEM, 20% FBS, and 2% antibiotic (penicillin/streptomycin, Capricorn). Cultures were maintained in standard culture conditions (37°C and 5% CO_2_). Once the cells reached 80–90% confluence, they were harvested with 0.25% (w/v) trypsin–EDTA (SAFC), counted, centrifuged at 700 *g* for 10 min, and resuspended in 1 mL of cryopreservation medium composed by 95% (v/v) FBS and 5% (v/v) dimethyl sulfoxide (DMSO) (MP Biomedicals). Cells were stored at −80°C for later use. In a second stage, eAD-MSCs were thawed in growth media (GM) with the following formulations: DMEM low glucose supplemented with 10% HPL, MPL, and FBS (reference condition) with 2 IU/mL sodium heparin (FU) to avoid clot formation (21) and 1% antibiotic. The cultures were maintained in standard culture conditions, and once the cultures of the different formulations reached confluence, the assays described below were performed. The study design is displayed in Figure 1B.

### 2.4 Cell proliferation

#### 2.4.1 MTT assay

1.6 × 10^3^ cells were seeded per well with 6 replicates per individual in 96-well plate (*n* = 7) in P_3_ and P_4_, and the evaluation was performed for 7 days. For this, a standard curve with 8 points was previously designed, with a seeding range from 0 to 6 × 10^4^ cells per well with 6 replicates; the plates were incubated for 24 h in order to extrapolate the concentration of cells through the equations of the curve (22). The total number of viable cells was determined by extrapolation from a calibration curve for each culture condition: FBS y = 6 × 10^−6^x + 0.0085, r^2^ = 0.9548; MPL y = 1 × 10^−5^x- 0.0393, r^2^ = 0.9859; HPL y = 1 × 10^−5^x- 0.0255, r^2^ = 0.9867 (y = optical density of the well; x = amount of cells). The assay followed the manufacturer’s recommendations (kit: M6494, Invitrogen), and the plate was read at 540 nm (Multiskan EX, Thermo Scientific).

#### 2.4.2 Cell doubling times

Passages 2–4 were selected for cell doubling times (DT) assays. Cells were seeded at a concentration of 5 × 10^3^ cells/cm^2^ in 6-well plates (*n* = 7). Every 72 h, the cells were washed with PBS, and the GM was changed. After 7 days, the cells were harvested with trypsin and counted in a Neubauer chamber using Trypan Blue 0.4% (Gibco) as the viability indicator. The DT and cell-doubling numbers (CD) for each passage were determined according to the following two formulae (23).

1. CD = ln(Nf/Ni)/ln(2)
2. DT = CT/CD
  CT the cell culture time, Nf the final number of cells, and Ni the initial number of cells.

#### 2.4.3 Colony-forming unit fibroblast assay

1 × 10^3^ cells were seeded per well in a 6-well plate (*n* = 7) for the three culture conditions in P_4_. The culture was maintained for 7 days, changing GM after 72 h. Then, the colonies obtained were fixed with cold methanol and stained with Giemsa (24). Macroscopic images of the plates were taken, and colonies were counted using Image J software.

### 2.5 Trilineage differentiation assay

The *in vitro* trilineage differentiation assay protocol was adapted from our previous study in other species (25, 26). Briefly, cells at P_3_ were seeded at a concentration of 1 × 10^4^/well in a 24-well plate (*n* = 7). When the cultures reached a cell confluence of 50–60% with GM of the different formulations, induction to different mesodermal lineages was initiated. For this purpose, cultures were maintained for up to 3 weeks with induction media for adipose, cartilage, and bone lineages supplemented with FBS, MPL, and HPL. Adipogenic: 500 μM 3-iso-butyl-1-methylxanthine (Sigma), 60 μM indomethacin (Sigma), 1 μM dexamethasone (Sigma), and 50 μg/mL insulin-transferrin-selenium A (Gibco). Chondrogenic: 1.7 mM ascorbic acid (Sigma), 10 ng/mL of TGF-β (Sigma), and 62.5 μg/mL insulin-transferrin-selenium A. Osteogenic: 60 μM dexamethasone, 10 mM β-glycerophosphate and 50 μM ascorbic acid. After cell induction, the cultures were fixed with 4% paraformaldehyde and stained to determine their *in vitro* differentiation with Oil Red O (Sigma), Alcian Blue stain (Biomedicals), or Alizarin Red S (Biomedicals), respectively. Every induction media was prepared with DMEM low glucose supplemented with 1% antibiotic and 2 IU/mL sodium heparin. Control cultures were maintained under the same GM conditions with each supplement.

### 2.6 Immune profile

#### 2.6.1 Immunophenotype

**Figure 2:**
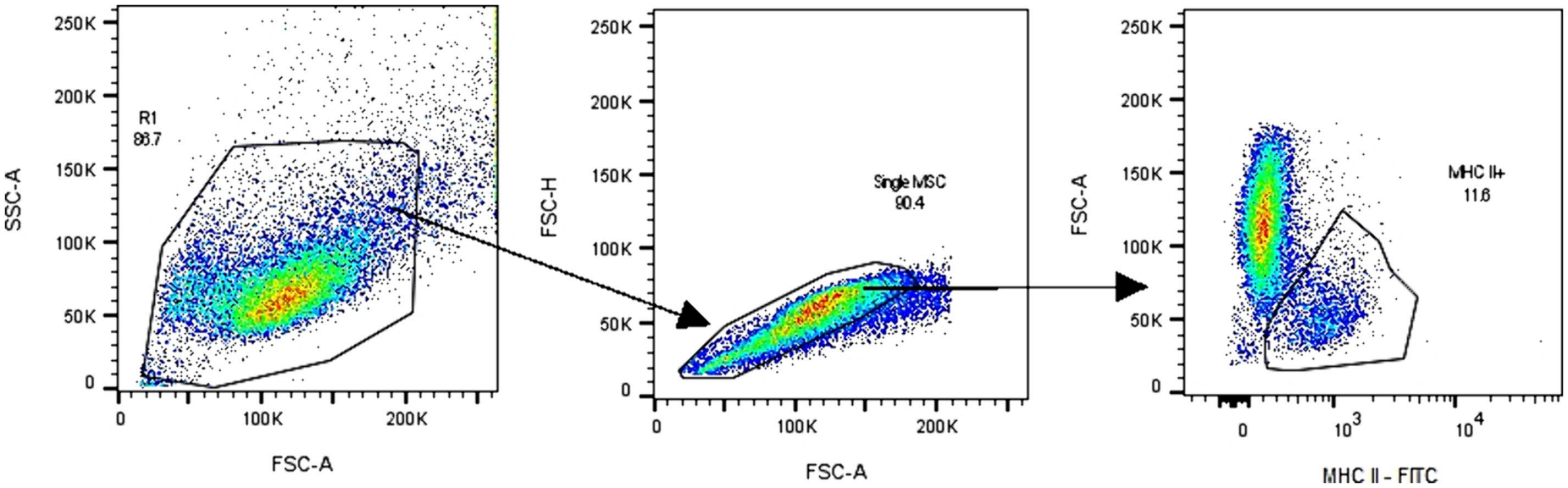
Flow cytometry analysis of adipose tissue-derived mesenchymal stromal cells (eAD-MSCs) involved a gating strategy, as depicted in the figure. First, the cells were identified and gated based on the FSC-A and SSC-A parameters, as observed in the corresponding dot plot. Subsequently, doublets were excluded by examining the FSC-H vs. FSC-A dot plot. Finally, the MHC II positive cells were gated, as demonstrated in the FSC-A vs. MHC II FITC dot plot. The data in the figure are shown as an example and representative of all samples analyzed.

The cell expression of major histocompatibility complex type II (MHC II) (*n* = 6) in P_3_ was studied by flow cytometry. For this, cells were seeded in a concentration of 5 × 10^3^/cm^2^ in T25 culture flasks. When the cells were 80% confluent, the culture medium was removed, and 5 mL of cold FACS buffer was added. FACS buffer consisted of PBS free of Ca^++^ and Mg^++^ containing 1 mM EDTA (ethylenediaminetetraacetic acid) and 0.5% of FBS. Cells were mechanically harvested, resuspended in FACS buffer, and centrifuged at 400 *g* for 5 min. The cells were resuspended in 100 μL FACS buffer, and 10 μL of the undiluted anti-MHC II antibody conjugated with fluorescein isothiocyanate (FITC) (clone CVS20, Invitrogen) was added. After 30 min incubation in the dark, cells were washed with FACS buffer and centrifuged at 400 *g* for 5 min. Finally, data was acquired on a BD FACS Canto II equipped with 488, 633, and 405 nm lasers. A minimum of 20,000 events per sample were acquired. Cells were gated based on FSC/SSC parameters, and the doublets were excluded (FSC-H vs. FSC-A). Moreover, MHC II positive MSCs cells were gated as shown in the dot plot FSC-A vs. MHCII FITC. The gating strategy, as described, is shown in Figure 2. Analysis was performed using BD FACSDiva Version 6.1.3 software or FlowJo™ V10.9 Software (BD Life Science).

#### 2.6.2 Gene expression profile

The gene expression profile was studied in eAD-MSCs (*n* = 4) in P_3_ cultured with MPL, HPL, or standard conditions with FBS. The cells were harvested with trypsin, then resuspended in culture medium and centrifuged for 5 min at 600 *g*. The supernatant was discarded, and 1 mL of TRIzol was added. Samples were dispensed into cryotubes and kept at −80°C until RNA purification.

Total RNA from eAD-MSCs was extracted using TRIzol reagent (Invitrogen) according to the manufacturer’s protocol. RNA concentration was measured using a NanoDrop ND-2000 spectrophotometer (NanoDrop Technologies, Wilmington, DE, United States). The RNA samples with A_260_/A_280_ and A_260_/A_230_ ratios in the range of 1.8–2.0 were used for cDNA synthesis. Total RNA (1 μg) was treated with 0.4 U DNase I (Invitrogen) to remove residual DNA and then reverse transcribed using Moloney murine leukemia virus (M-MLV) reverse transcriptase (Invitrogen) and Random primers and RNaseOUT (both Invitrogen) in a final 20-μL reaction mixture.

Following retrotranscription, quantitative PCR for MHC-I, MHC-II, IL-6, IL-10, and TNF-α mRNA was conducted using QuantiTect^®^ SYBR^®^ Green PCR Kit (Qiagen) in a Rotor-Gene (QIAGEN) thermocycler. The glyceraldehyde-3-phosphate dehydrogenase (GAPDH) was used as a housekeeping gene. Primer details, accession numbers for mRNA sequences, and amplicon sizes are shown in Table 1 and have been previously validated and used in other studies, as well as the RT-qPCR methodology (27, 28). Cycle program was as follows: initial incubation 15 min at 95°C; followed by 40 cycles of 15 s at 95°C, 1 min at 60°C with data acquisition; and final a melt curve with a ramp from 60 to 95°C at 1°C/s. Melt curve analysis was used to identify and exclude reactions with alternative amplicons. Each primer pair was validated, and its efficiency was calculated using a standard curve of six threefold serial dilutions of a representative sample of pooled cDNA (data not shown). Gene expression levels were determined by the 2^−ΔΔCt^ method as previously described (29), using the GAPDH gene as the normalizing gene (ΔCt = Ct _gene of interest_ − Ct _GAPDH_) and standard condition (FBS) as the calibration condition (DDCt).

**Table 1:** Genes analyzed by real time quantitative polymerase chain reaction (RT-qPCR).

Gene	Accession number	Primer sequence (5′–3′)	Amplicon size
Antigen receptors			
MHC-I	AB525081	F: CGTGAGCATCATTGTTGGC	92
		R: TCCCTCTTTTTTCACCTGAGG	
MHC-II	NM_001142816	F: AGCGGCGAGTTGAACCTACAGT	172
		R: CGGATCAGACCTGTGGAGATGA	
Interleukins and cytokines			
IL-6	EU438770	F: AACAGCAAGGAGGTACTGGCA	95
		R: CAGGTCTCCTGATTGAACCCA	
IL-10	EU438771	F: GACATCAAGGAGCACGTGAACT	140
		R:TGGAGCTTACTGAAGGCACTCT	
TNF-α	EU438779	F: CATGTTGTAGCAAACCCCCAA	125
		R: TACAGCCCATCCAATGGTACC	
Housekeeping			
GAPHD	NM_001163856	F: GGCAAGTTCCATGGCACAGT	128
		R: CACAACATATTCAGCACCAGCAT	

GenBank accession numbers of the sequences used for primers design. Primer sequences (F, Forward and R, Reverse) and amplicon length in base pair (bp). Genes were grouped in agreement with the functions and implications of encoded molecules to facilitate the posterior analysis: Antigen receptors, interleukins and cytokines, housekeeping.

#### 2.6.3 Immunomodulatory cytokine quantification

Quantification of IL-6, IL-10, and TNF-α on culture supernatant of each of three conditions (*n* = 7) was performed using DuoSet ELISA kits (DY1886, DY1605, and DY1814, R&D Systems). Samples were collected from different cultures conditions and stored at −80°C. For the ELISAs, the samples were thawed and used undiluted for concentration determination. Subsequently, the manufacturer’s recommendations were followed to quantify each cytokine. Data were read using a spectrophotometer at an optical density of 450 nm (Multiskan EX, Thermo Scientific).

### 2.7 Statistical analysis

Data were expressed as mean ± standard error of the mean (SEM) or median interquartile range (Q25; Q75) and analyzed using the Lilliefors normality test. Parametric or non-parametric statistics were employed based on the distribution of the data. Statistical analysis involved using ANOVA and Tukey parametric test as post-hoc for immunophenotype and Kruskal-Wallis test and Dunn’s test as post-hoc for gene expression. The analysis of samples in relation to concentrations of platelets, leukocytes, and GFs variables was performed with generalized linear mixed models, taking into account fixed variables (treatment) and random effects (individual). For cell proliferation and cytokines in the supernatant, the fixed effects were condition and passage, and the random effect was the individual. All statistical analyses were conducted using GraphPad Prism 8 software or R (version 4.1.2), and a *p*-value of <0.05 was considered statistically significant.

## 3 Results

### 3.1 Characterization of allogeneic PL

**Figure 3:**
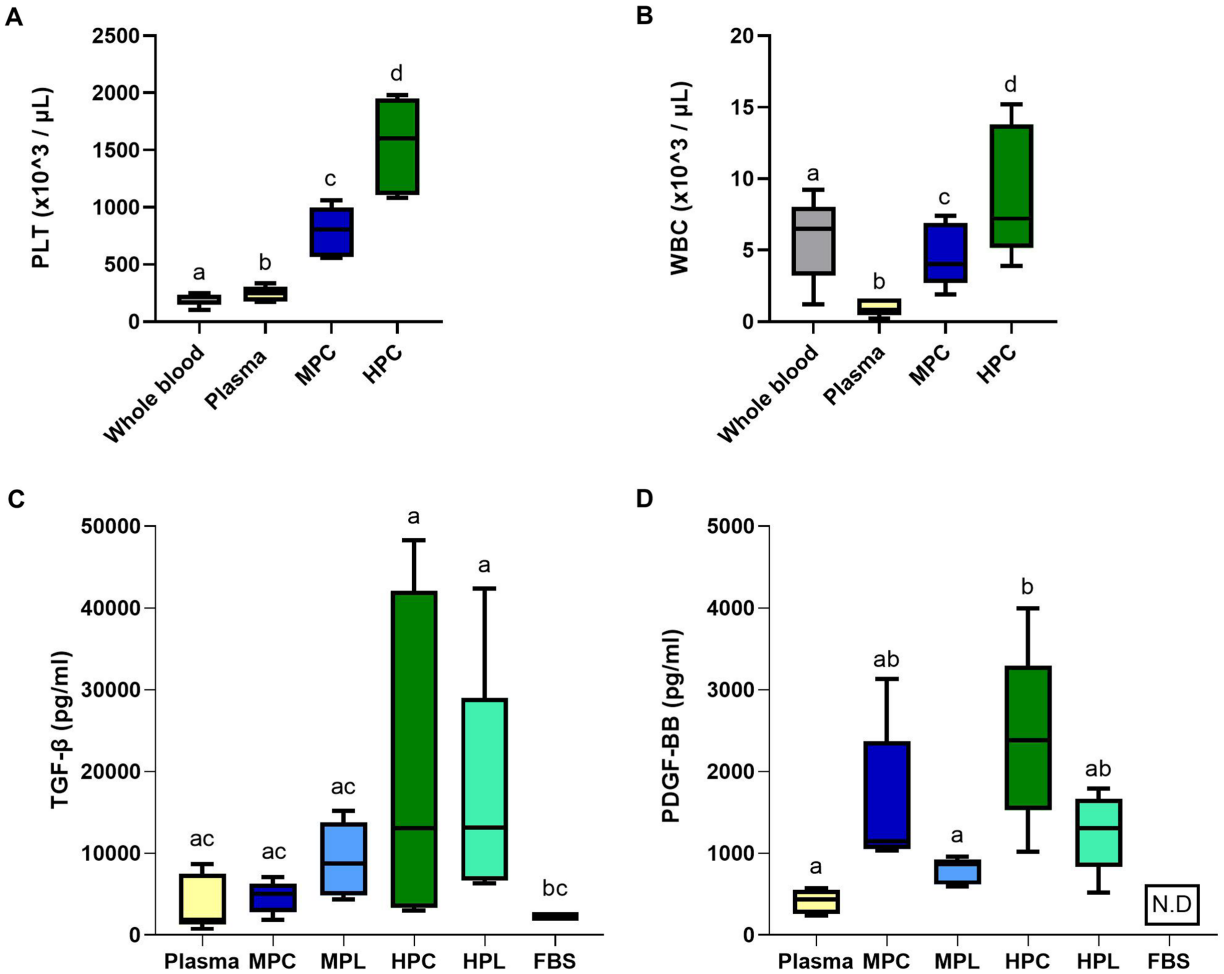
Hematological values and growth factor concentration for the different platelet lysate formulations. Boxplots show the differences of platelet (PLT; **A**), white blood cell (WBC; **B**) for the medium-concentration platelet lysate (MPL) and high-concertation platelet lysate (HPL) formulation prior to freeze/thaw cycle, called medium platelet concentrate (MPC) and high platelet concentrate (HPC). For the measurement of growth factors, differences in transforming growth factor-beta (TGF-β; **C**) and platelet-derived growth factor-BB (PDGF-BB; **D**) concentrations for different PL formulations are shown. Results are expressed as median (interquartile range Q25; Q75). Different lowercase letters denote significant differences between groups; *p* < 0.05. Data were obtained from *n* = 5 horses. N.D, not detection.

A higher platelet concentration of 3.3× was achieved for MPC (pre-freeze/thaw MPL formulation) with 805 (567; 996) × 10^3^/μL and 6.5× for HPC (pre-freeze/thaw HPL formulation) with 1,603 (1,108; 1952) × 10^3^/μL relative to the baseline plasma value of 247 (176; 307) × 10^3^/μL, the WB value of 217 (147; 238) × 10^3^/μL. Significant differences were found among all the evaluated blood derivative formulations compared to WB (Figure 3A). Additionally, there was a significant increase in leukocytes for HPC with 7.2 (5.1; 13.8) × 10^3^/μL compared to plasma with 0.8 (0.4; 1.6) × 10^3^/μL and WB with 6.5 (3.2; 8.0) × 10^3^/μL. In contrast, MPC with 4.2 (2.7; 6.9) × 10^3^/μL showed a significant decrease when compared to WB (Figure 3B). The efficiency to obtain MPC was 12 and 5.7% for HPL in relation to the total volume of blood required for its preparation. On the other hand, when analyzing the GFs, there was a higher concentration of TGF-β for MPL with 8.7 (4.8; 13.7) × 10^3^ pg/mL and HPL with 13 (6.7; 29) × 10^3^ pg/mL relative to the basal value of plasma with 1.8 (1.2; 7.5) × 10^3^ pg/mL, but no difference was found between formulation (Figure 3C). Furthermore, we found significantly higher concentrations in HPL and HPC compared to FBS, showing the last one a concentration of 2.3 × 10^3^ pg/mL. In the case of PDGF-BB, higher concentrations were found in HPC and MPC (trend *p* = 0.052) compared to plasma. There was no significant reduction in the transition from PC formulations to PL, except when comparing HPC to HPL, where a slight decrease was observed (trend *p* = 0.065). The final concentrations of PDGF-BB were 0.8 (0.6; 0.9) × 10^3^ pg/mL for MPL, 1.3 (0.8; 1.6) × 10^3^ pg/mL for HPL, and 0.4 (2.5; 5.4) × 10^3^ pg/mL for plasma. For FBS, this GF was not detected (Figure 3D). The parameters of the statistical models can be found in Supplementary Tables S1–S4.

### 3.2 Cell proliferation

**Figure 4:**
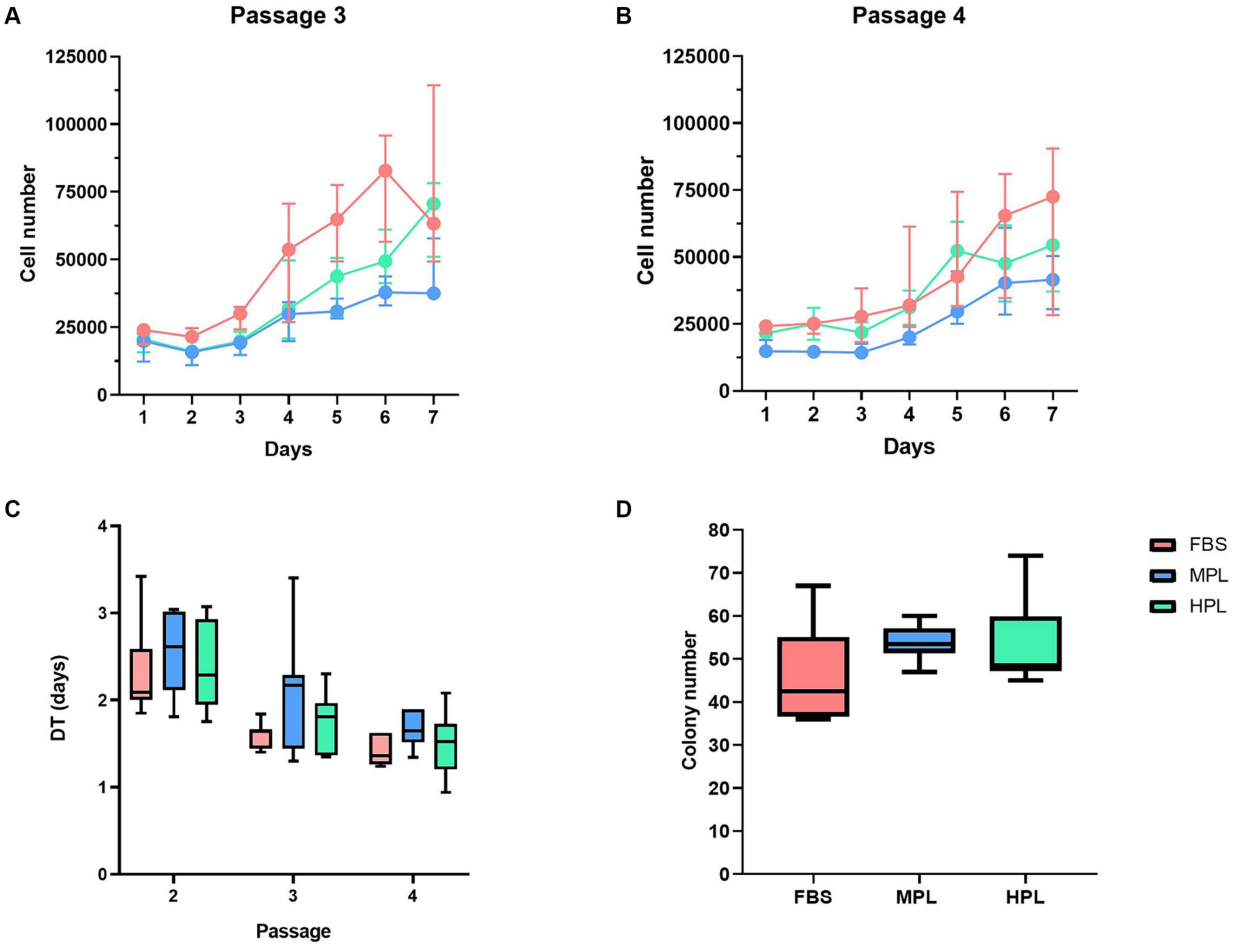
Growth of equine adipose tissue-derived mesenchymal stromal cells (eAD-MSCs) cultured with different formulations of platelet lysate as supplement. The three conditions were evaluated for 7 days using the MTT assay in passages P_3_ **(A)** and P_4_ **(B)** of cultures supplemented with fetal bovine serum (FBS) as reference condition, medium-concentration platelet lysate (MPL) and high-concentration platelet lysate (HPL) formulations. Boxplots show the differences in each cell passage by determining cell doubling times (DT; **C**). Likewise, the clonogenic capacity of **(D)** cells cultured in the same conditions was evaluated in P_4_. Results are expressed as median (interquartile range Q25; Q75). Data obtained from eAD-MSC from *n* = 7 donors.

In all culture conditions, monolayer growth, cells with fibroblastic morphology, adherence to the plastic, and the capacity to form colonies were observed (Supplementary Figure S1). We observed the following results when we studied cell proliferation using different assays. For the MTT assay in P_3_ and P_4_, the highest proliferation ability was observed in FBS, followed by HPL and MPL, with significant differences observed among all conditions (Figures 4A,B). These differences remained consistent across the two passages studied. Furthermore, significant differences were observed for all three conditions when comparing passages, indicating a passage effect on proliferation ability. When assessing proliferative ability through DT determination between P_2_ and P_4_, no significant differences were found when comparing the different culture supplements within each passage (Figure 4C). However, the evaluation of the passage effect within each condition revealed a decrease in DT values in P_3_ and P_4_ compared to P_2_. Finally, the colony-forming capacity evaluated in P_4_ was similar across conditions, with no significant differences observed. The counts obtained in the colony-forming unit fibroblast assay were 42.5 (36.7; 55) for FBS, 53.5 (51.5; 57) for MPL, and 48.5 (47.2; 59.7) in P_4_ (Figure 4D). The parameters of the statistical models can be found in Supplementary Tables S5–S7.

### 3.3 Trilineage differentiation

**Figure 5:**
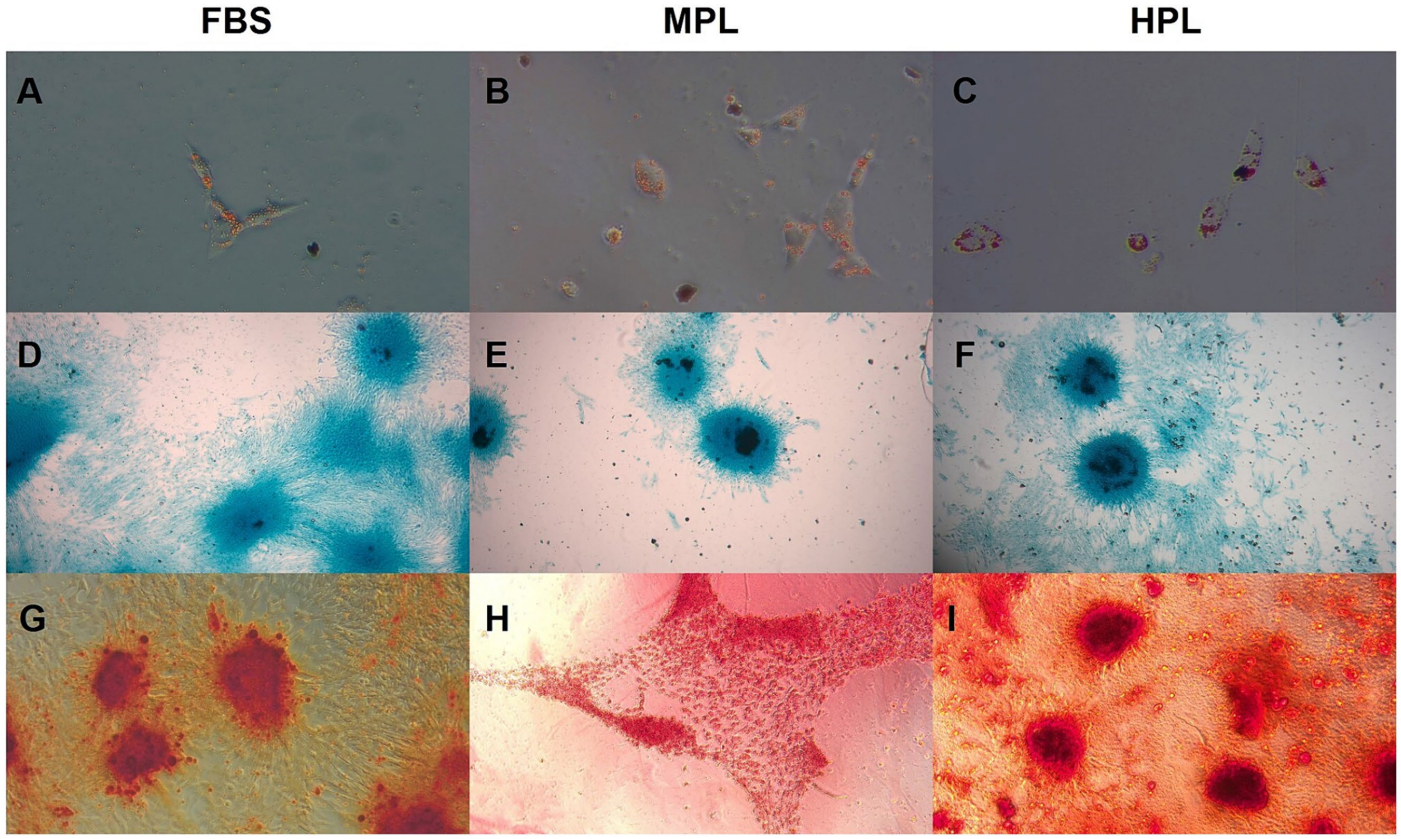
*In vitro* tridifferentiation assay of equine adipose tissue-derived mesenchymal stromal cells (eAD-MSCs) cultured with fetal bovine serum (FBS), medium-concentration platelet lysate (MPL) and high-concentration platelet lysate (HPL). **(A–C)** Adipose lineage stained with Oil Red O dye, showing vacuoles (40×). **(D–F)** Cartilaginous lineage stained with Alcian Blue stain, demonstrating the extracellular matrix (4×). **(G–I)** Bone lineage stained with Alizarin Red S stain, indicating the mineralized matrix (10×). Data obtained from eAD-MSCs from *n* = 7 donors.

The *in vitro* multipotency capacity of eAD-MSCs cultured with PL as a supplement was tested. In the adipose lineage, intracytoplasmic vacuoles were stained red with Oil Red O (Figures 5A–C). Similarly, an affinity for the bluish Alcian Blue dye was observed in the chondrogenic lineage, indicating the synthesis of glycosaminoglycans as part of the extracellular matrix (Figures 5D–F). The osteogenic lineage showed mineralized matrix deposition due to the affinity for the reddish Alizarin Red S stain (Figures 5G–I). Negative controls did not show an affinity for any of the stains used.

### 3.4 Immune profile

#### 3.4.1 Immunophenotyping

**Figure 6:**
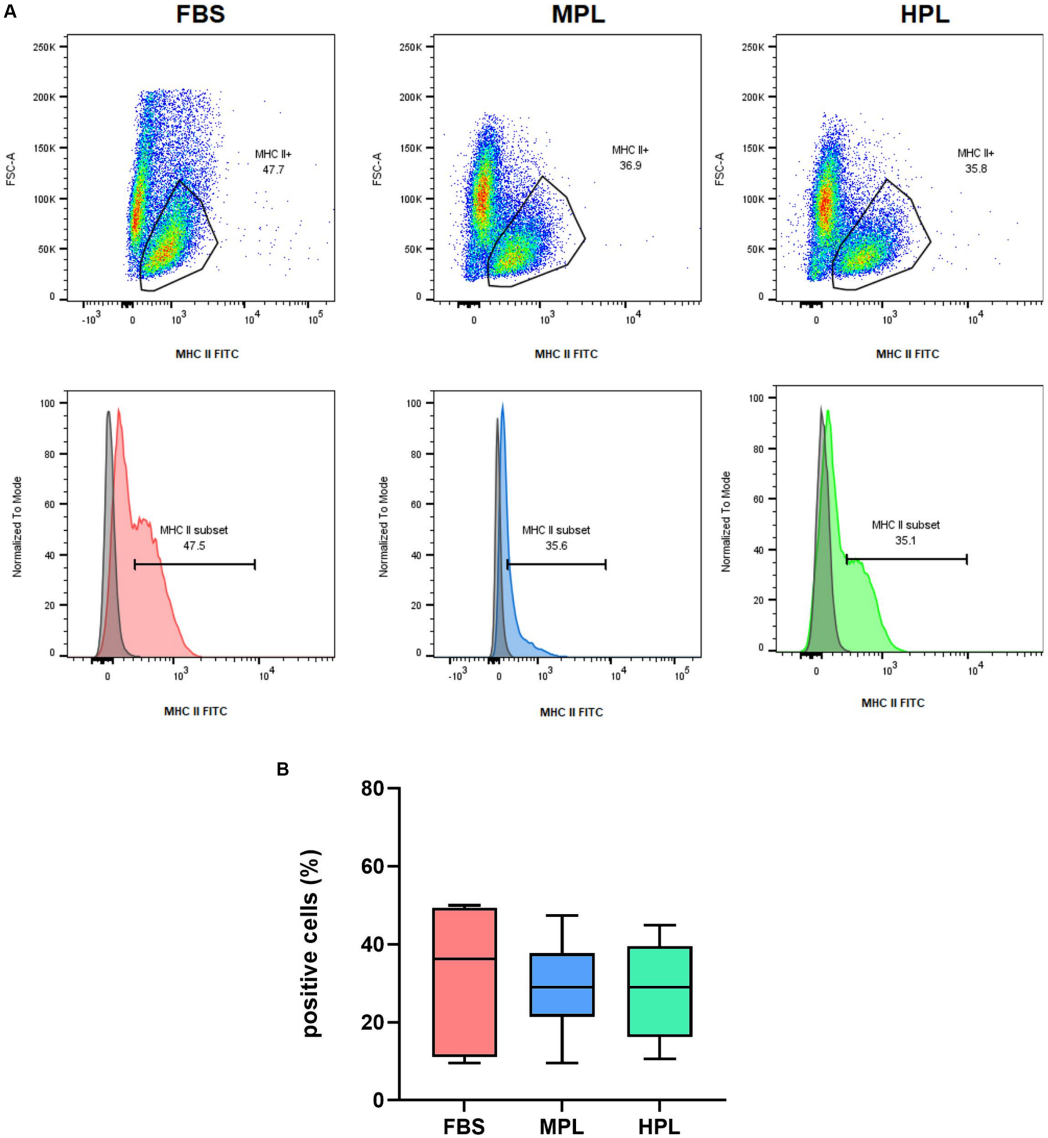
Flow cytometry analysis was conducted on equine adipose tissue-derived mesenchymal stromal cells (eAD-MSC) that were cultured with fetal bovine serum (FBS), medium-concentration platelet lysate (MPL), or high-concentration platelet lysate (HPL). The cells were mechanically detached and stained for MHC class II surface expression. Representative examples of FSC-A vs. MHC-II FITC dot plots and overlaid histograms of the surface MHC-II staining and their respective unstained control of eAD-MSCs for each condition were presented **(A)**. Boxplots displaying the percentage of MHC-II positive cells **(B)**. Results are expressed as median (interquartile range Q25; Q75). The data analyzed were obtained from eAD-MSC from *n* = 6 donors in passage 3.

MHC II expression on eAD-MSCs was assessed by flow cytometry. Some dispersion was observed among cells cultured with FBS, MPL, and HPL, with a median of 36 (11; 49) %, 29 (21; 38) %, and 29 (16; 40) % of MHC II^+^ cells, respectively (Figure 6). No significant differences were observed among groups. No significant differences were observed among the three assayed conditions regarding MHC II Mean Fluorescence Intensity (data not shown).

#### 3.4.2 Genes expression

**Figure 7:**
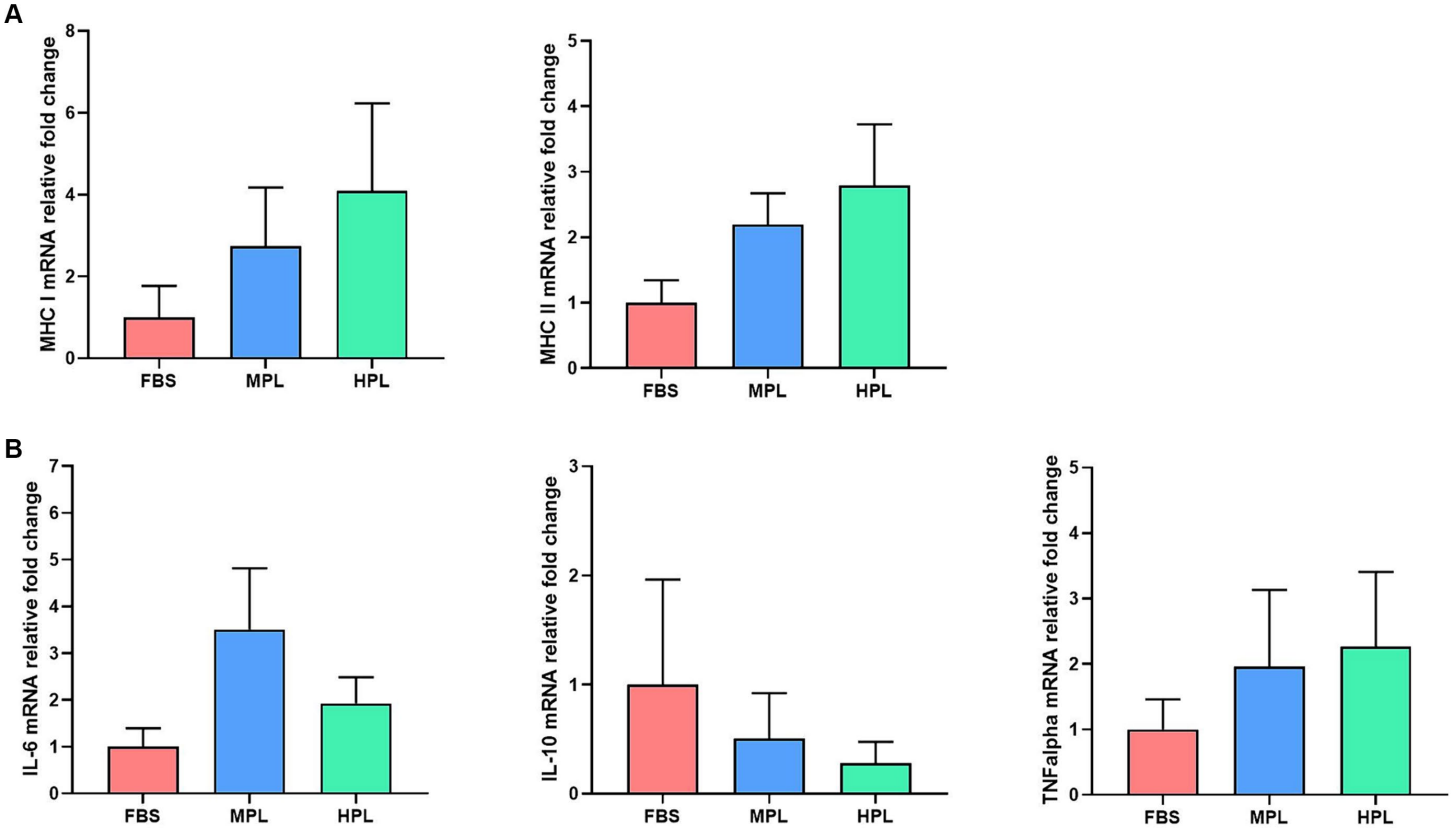
Gene expression of equine adipose tissue-derived mesenchymal stromal cells (eAD-MSCs) in three culture conditions: fetal bovine serum (FBS), medium-concentration platelet lysate (MPL), and high-concentration platelet lysate (HPL). Bars represent the mean ± SEM of the relative mRNA levels of genes encoding immunogenic-related molecules **(A)** and immunomodulator-related molecules **(B)**. For each sample, mRNA levels were normalized to the GAPDH gene and expressed as relative levels compared to the FBS condition. Data were obtained from eAD-MSC from *n* = 4 donors.

The immunogenic markers MHC I and II showed similar levels of expression in every culture condition without significant differences between them. However, the results indicate that the expression in the cultures with PL showed a slight increase for both genes compared to the culture with FBS (Figure 7A). A similar trend was observed for the expression of the cytokines IL-6 and TNF-α; both PL and FBS cultures exhibited similar expression levels (Figure 7B). Conversely, PL cultures showed a slight decrease in the expression level of IL-10 compared to the reference culture, although without significant differences (Figure 7B).

#### 3.4.3 Immunomodulatory cytokine quantification

**Figure 8:**
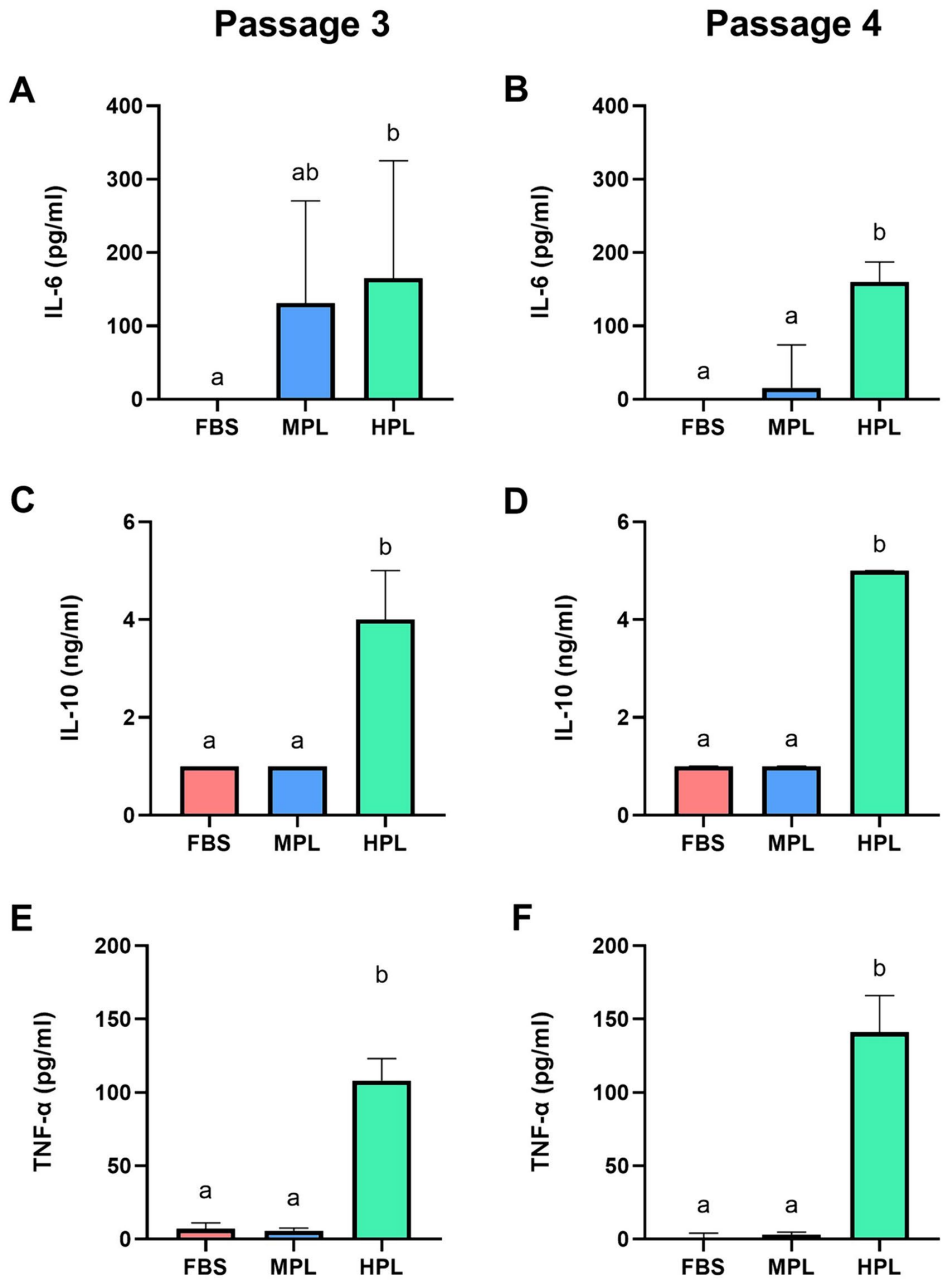
Concentration of immunomodulatory molecules in cultured equine adipose tissue-derived mesenchymal stromal cells (eAD-MSCs) cultured with different formulations of platelet lysate as a supplement. Boxplots show the concentrations of interleukin 6 (IL-6; **A,B**), IL-10 **(C,D)** and tumor necrosis factor-alpha (TNF-α; **E,F**) from culture supernatant supplemented with fetal bovine serum (FBS), medium-concentration platelet lysate (MPL) and high-concentration platelet lysate (HPL) at passage 3 and 4. Results are expressed as median (interquartile range Q25; Q75). Different lowercase letters denote significant differences between groups; *p* < 0.05. Data obtained from eAD-MSC from *n* = 7 donors.

The concentration of various immunomodulatory cytokines was determined in successive passages (P_3_ and P_4_) in the culture supernatant of eAD-MSCs under different conditions. In the MPL culture supernatant, an increase in IL-6 was found compared to FBS (trend *p* = 0.051) in P_3_, which decreased in P_4_ (Figures 8A,B). This decrease was significant when comparing the MPL condition among these passages. No differences were found with FBS nor between passages for this condition for IL-10 (Figures 8C,D) and TNF-α (Figures 8E,F). Conversely, the HPL supernatant cultured presented significantly higher concentrations of IL-6, IL-10, and TNF-α than MPL and FBS. A significant variation was determined regarding the passage effect, with a decrease in IL-6 and an increase in TNF-α in P_4_ for this condition. The parameters of the statistical models can be found in Supplementary Tables S8–S10.

## 4 Discussion

In this study, three main findings were observed. First, when studying the proliferation ability of eAD-MSCs, we observed comparable results between the two PL formulations despite a difference in the platelet concentration of HPL compared to MPL. This indicates that both were suitable for *in vitro* propagation. Additionally, the results were comparable to the standard condition (FBS). Second, when exploring the immunogenic profile of MHC I and MHC II through flow cytometry and gene expression, a similar expression of MHC surface markers was observed for cultures with PL, comparable to those with FBS. At the gene expression level, there were slight increases compared to FBS, indicating that eAD-MSCs cultured with PL have a slightly different profile compared to the standard condition. Third, when exploring the immunomodulatory profile through gene expression and cytokine concentration, a slight increase in the expression of IL-6 and TNF-α and a reduction of IL-10 were observed compared to the profile of cells cultured with FBS. Furthermore, when the concentration of these cytokines in the culture supernatant was determined, an increase in IL-6, IL-10, and TNF-α was found for the HPL condition and only IL-6 for MPL compared to FBS. This indicates that the PL formulation significantly modified the cytokine microenvironment in the cell culture.

Blood parameters were evaluated during the elaboration process of the MPL and HPL supplements. The platelet counts obtained indicated that there were 805 and 1,603 × 10^3^ PLT/μL for MPL and HPL, respectively. When comparing our results with the literature, we found a significant heterogeneity of platelet concentrations. Other authors describe lower concentrations, such as 357 × 10^3^ (17), 484 × 10^3^ (10), 591 × 10^3^ (19), 1,000 × 10^3^ PLT/μL (12, 15), or even higher at 2,000 × 10^3^ PLT/μL (30). The use of different methods for obtention can explain these variations in platelet concentration. In our work, we used the double centrifugation method previously described (15). We took the volume as a reference, concentrating 5 (MPL) or 10 (HPL) folds of the initial plasma volume, simplifying its elaboration, similar to reports from other authors (31) and previous descriptions of our group (19). In contrast, some works set the concentration at 1,000 × 10^3^ PLT/μL for equine PL (12, 15), which could be considered as a reference adopted from the recommended value for humans (9, 18). However, despite the significant variability, satisfactory results are described for the *in vitro* propagation of eMSCs with values below the reference concentration, as observed in the results using MPL.

When the platelet:leukocyte ratio was determined, we observed that it was similar between formulations, obtaining 170:1 for MPL and 171:1 for HPL. These results contrast with descriptions of other authors who obtained a different ratio of 1,175:1 (12) and 1,002:1 (19) for concentrated PL, indicating a higher number of leukocytes in our formulations, suggesting that we can use leukocyte-rich platelet-rich plasma (L-PRP) to formulate the PL when compared with the literature. Nevertheless, the effect of platelet:leukocyte ratio on MSC cultures is not elucidated in the literature (10, 12, 19). Regarding the quantification of leukocytes, we obtained higher values than those described in the literature, with 4.2 and 7.2 × 10^3^ WBC/μL for MPL and HPL, respectively. We have previously reported 590 WBC/μL in equine PL (19). Thus, we could speculate that the differences may be due to manipulation and/or individual variation in equine donors, suggesting that these variations in the methodology used may not be sufficiently reproducible and thus be considered a limitation to its use (10). On the other hand, authors using the apheresis method showed lower counts of 150 WBC/μL (17) because leukocyte reduction is performed mechanically by separating the cellular components in the extraction process. Regardless, the effect the WBC count in equine PL as a culture supplement could have on the properties of MSCs is not determined.

In our work, the concentration of TGF-β and PDGF-BB was determined because these GFs positively influence MSC proliferation (6, 18). Overall, we found an increase in both GFs for MPL and HPL compared to plasma and FBS values, which is in agreement with the equine PL literature (10, 11). We obtained a TGF-β concentration of 8.7 and 13 ng/mL for MPL and HPL, respectively. Other authors describe lower values for the HPL formulation, such as 3.9 ng/mL (10, 20) and 7.3 ng/mL (17), and also higher values, such as 24.5 ng/mL (12). On the other hand, PDGF-BB values were 0.8 and 1.3 ng/mL for MPL and HPL, respectively, and other authors reported higher values, such as 3.6 (17), 3.7 (10, 20), and 5.2 ng/mL (12). A slight decrease in PDGF-BB was observed when comparing the MPL and HPL formulations with MPC and HPC. This could be attributed to various factors such as increased manipulation and thermal changes (freeze/thaw cycle) of the sample, which involves transitioning from a PC to PL, potentially explaining the observed phenomenon. This coincides with the decrease in this GF described for canine PL (20), which contradicts the reported findings in equine species showing a slight increase in PDGF in PL compared to PC (10).

These variations in the concentration of both GFs may be due to different numbers of freeze/thaw cycles in the plasma activation process. In our case, only one freeze/thaw cycle was used, coinciding with previous reports (11, 12, 15) but contrasting with others (10, 16, 17). Therefore, it would be important to study which are the optimal concentrations of TGF-β and PDGF-BB in the PL formulation since these could be a standardized value to use in culture, instead of the platelet value, since the same number of platelets does not guarantee the same GFs concentration (9, 10, 32).

For the isolation of eMSCs in this study, GM supplemented with FBS and cryopreservation was used. After thawing the cells, the supplement was switched to formulations containing PL for proliferation, multipotency, and immune profile assessments. This strategy aligns with most reported studies that evaluate PL as a supplement for eMSC cultures (10, 12, 14, 16). Conversely, other studies have used PL from the onset of eMSC isolation (11, 15). In both strategies, replacing FBS with PL as a supplement achieved *in vitro* propagation of equine cells with comparable results across studies. However, it is not known how the characteristics of eMSCs may be influenced if PL is used at the start of culture, during isolation, or afterward.

*In vivo* propagation of eAD-MSCs was achieved using PL as a supplement, coinciding with previous descriptions (10, 30). Other authors describe PL as a culture supplement comparable to FBS for the expansion of cells from another source in equines, such as bone marrow-derived MSCs (BM-MSCs) (11, 16). We found discrepant results when evaluating the proliferation ability using the MTT and DT assays. On one hand, the MTT assay indicated a higher growth rate in FBS compared to PL conditions. Furthermore, HPL showed a higher rate than MPL, which could be expected due to the higher concentration of GFs in HPL. On the other hand, through DT determination in successive passages, no significant differences were found between conditions for each passage studied. There is limited research on the proliferation rate in cultures with PL in successive passages for eMSCs.

In the case of MTT assay evaluation, our group’s previous findings showed a higher proliferative capacity using concentrated PL (19), which disagrees with our current results. However, when comparing proliferation rates through DT, our results align with most of the literature, which does not find differences between cultures with PL and FBS (10, 15, 16). Nonetheless, these results also differ from our previously published findings, which reported a lower DT for the PL condition compared to FBS in P_3_. One reason for the discrepancies between our current results and previous assays from our group using MTT and DT could be the concentration of PL used, which was 10% (v/v) relative to the total volume of the culture medium. This concentration is consistent with previous research (10, 16). However, it differs from our earlier results, which showed a higher growth rate using concentrated PL but in a concentration of 20% (v/v) (19). This difference can be explained by the effect of supplement concentration on proliferation ability, as a dose-dependent relationship between supplement and cell proliferation has been demonstrated. It has been indicated that the optimal range for use is between 10 and 30% (12, 30).

*In vitro* multipotency was evaluated through the classical trilineage differentiation assay to adipose, cartilage, and bone lines as recommended by the International Society for Cell and Gene Therapy (ISCT) (33, 34). We succeeded in testing multipotency in all three induced cell lineages for both PL conditions. This is in agreement with other authors who have achieved these results for PL-cultured eMSCs (10, 16) in other domestic species (20) and humans (18). However, not all studies using PL as a supplement for eMSCs culture perform this classical assay for all recommended mesodermal lineages (11, 15, 30). Therefore, sharing characterization criteria of multipotent MSCs is important to allow comparison between studies. One limitation of this study was that the *in vitro* multi-differentiation capacity between conditions was not quantitatively compared. Other studies have quantitatively explored the multipotentiality of eMSCs cultured with PL and have described results comparable to FBS (10, 20). Another limitation of this study in the characterization of eMSCs was the lack of cell immunophenotyping. It is recommended that the expression of certain surface markers established for human MSCs be determined (33).

To discuss the immune profile of the cells, we will first address the immunogenic profile and then the immunomodulatory profile. The gene expression of MHC I and II was evaluated, and for MHC II also by flow cytometry as a surface marker. A similar expression was found in the three culture conditions, coinciding with the existing literature where a similar expression profile is found between cells supplemented with PL in relation to FBS (10, 14, 16) and with precedents published by our group (19). Although a slight (not significant) increase in the expression of both genes was observed in cells cultured with PL compared to FBS, it is hypothesized that the high concentration of leukocytes present in both PL formulations could have created a pro-inflammatory microenvironment that altered the expression of these immunogenic markers (35).

We obtained, by flow cytometry, a high proportion of MHC II expressing cells, with a median of 29% positive cells in both MPL or HPL culture conditions. Other works evaluating PL as a supplement to eMSCs describe <10% expression (10, 11, 16). The literature describes eMSCs with both high or low expression of this marker under basal conditions cultured with FBS in the absence of *in vitro* pro-inflammatory stimuli (3, 36, 37). This donor-dependent variability could explain the discrepancies between the literature and our results. The type of antibody clone used for MHC II immunolabeling may explain these differences. We used a monoclonal antibody directly conjugated to FITC (clone CVS20), the same clone used by Hagen et al. (10), though they found a lower proportion of MHC II positive cells. On the other hand, different MHC II antibody clones, like EqT2 (16) or cz11/IgG1 (11), have been used in the literature. Besides, some authors included a viability dye in the flow cytometry analysis (10, 36), while others, like us, did not (11, 16, 37). We consider that this could influence the results of our analysis of MHC II positive cells. The cell source and passage assessed could also have effects. In our work, eAD-MSCs were assessed at P_3_, while in the work of Even et al. (11) and Naskou et al. (16), BM-MSCs were assessed at P_3_ and P_4_, respectively. Finally, the heterogeneity of MHC II expression at the basal level of eMSCs we observed in our results is not new in the literature. The presence of this immunogenic antigen in eMSCs cultured in PL could promote their immunological rejection in the hypothetical case of their application with MHC-mismatched, which is why it is becoming increasingly important to apply MSCs with MHC-matched (35, 38).

Regarding the immune profile of eAD-MSCs, gene expression and the presence of immunomodulatory molecules such as IL-6, IL-10, and TNF-α were compared among the different culture conditions. At the gene expression level, no significant differences were found in both media types with PL compared to the reference condition. However, a slight increase in IL-6 and TNF-α and a slight decrease in IL-10 were observed for both PL conditions compared to FBS. There is limited background information on the gene expression profile with immunological markers in eMSC cultured with PL. Our group has previous experience with this type of evaluation, finding a significantly high expression level of IL-6 in equine BM-MSCs (19). The origin of the MSCs could explain the differences with the work mentioned above, as cells isolated from bone marrow were analyzed in our previous study, whereas in this work, cells of adipose tissue origin were analyzed. It has been observed that the source of MSCs can modify the gene expression profile (39).

Previous reports observed, when evaluating the gene expression profile of eMSCs receiving an *in vitro* pro-inflammatory stimulus with TNF-α and INF-γ, an upregulation of IL-6 but not of IL-10 when compared to unstimulated cells (28, 40), which is similar to our results despite not finding significant differences. Therefore, under our conditions, we could hypothesize that the cells showed a modified immunogenic and immunomodulatory profile compared to the reference condition. This suggests they might be in a pro-inflammatory environment due to these slight increases in key markers for MSCs. However, one of the limitations of this study could be the lack of gene expression monitoring over the culture time. Tracking gene expression over time could provide more insights, as not only the stimuli to which MSCs are exposed alter their immune profile, but the post-exposure time also affects it (41, 42).

Regarding the quantification of IL-6, IL-10, and TNF-α in the culture supernatant of eAD-MSCs, we found significantly higher concentrations of the three cytokines for the HPL condition, and only IL-6 was significantly higher for MPL compared to the reference condition (FBS). These results lead to the hypothesis that the use of PL in culture could simulate an *in vitro* pro-inflammatory stimulus and thus could enhance the immunomodulatory capacity of MSCs (5, 11, 14). References on the measurement of immunomodulatory cytokines in MSCs culture with PL are scarce. Even et al. (11) found no significant difference in the concentration of IL-6, IL-10, and TNF-α in the culture supernatant of equine BM-MSCs without stimulation, comparing PL and FBS supplementation, which disagrees with our results where we found high concentrations, especially in the HPL formulation. These discrepancies could be explained by the fact that the platelet concentration in our HPL was higher than in the PL they used, and this could potentially concentrate the immunomodulatory cytokines. On the other hand, there is a high individual variability in the equine donors used to elaborate the PL, which may influence the final concentration in the PL pool. Another important difference is the time in which the sample was taken to measure cytokines in the supernatant. In our case, it was taken after 72 h of culture instead of 48 h (11).

In the study by Moellerberndt et al. (13), they measured the concentration of these cytokines directly on the PL and observed that there was a significant individual variation in the concentration and described animals with high concentration and others with no detectable cytokines. The same group recently evaluated the cytokine concentration in the culture supernatant of eAD-MSCs with PL media and found higher concentrations of TNF-α and IL-10 in the PL media than in FBS media (14). Additionally, when comparing the basal value of PL media with the culture supernatant after a 72 h incubation, they found that the level of TNF-α and IL-10 remained stable. These results are consistent with ours, as we detected higher concentrations of these cytokines in the HPL media supernatant than in FBS media. However, a limitation of this study is that the baseline cytokine levels prior to incubation with MSCs were not determined. Therefore, we cannot confirm if there was a change from the original value of the PL medium. Nevertheless, we can hypothesize that there were no high variations based on recent studies showing stability in these cytokines (14).

In conclusion, the MPL and HPL culture supplement formulations contained concentrations of GFs similar to each other and higher than those present in FBS. Both supplements allowed *in vitro* culture of eAD-MSCs with proliferative, clonogenic, and multipotent capacity comparable to the reference medium. However, higher concentration of IL-6, IL-10, and TNF-α in the culture supernatant were observed for the HPL condition compared to those cultured in MPL and FBS. This indicates that the culture microenvironment is modified and could potentially impact the immunomodulatory profile of eAD-MSCs. Nonetheless, further studies are needed to investigate the effect of this variation in cytokines on the safety and therapeutic efficacy of MSCs, especially in immune-mediated pathologies.

## Data availability statement

The datasets presented in this study can be found in online repositories. The names of the repository/repositories and accession number(s) can be found in the article/Supplementary material.

## Ethics statement

This study was carried out, in strict accordance with the recommendations of the Honorary Commission for Animal Experimentation (CHEA) of Uruguay under approval identification CEUA-FVET 1097 and 1625. The study was conducted in accordance with the local legislation and institutional requirements.

## Author contributions

KY: Conceptualization, Data curation, Formal analysis, Funding acquisition, Investigation, Methodology, Project administration, Writing – original draft. GÁ: Writing – original draft, Methodology, Investigation, Formal analysis, Data curation. ARo: Writing – review & editing, Writing – original draft, Supervision, Methodology, Formal analysis. ARi: Writing – review & editing, Writing – original draft, Supervision, Methodology, Formal analysis. SC: Methodology, Writing – original draft. ME: Methodology, Writing – original draft. AA: Writing – review & editing, Writing – original draft, Supervision, Methodology, Conceptualization. GS: Data curation, Formal analysis, Visualization, Writing – review & editing.

## Supplementary material

Supplementary FIGURE S1 Equine adipose tissue-derived mesenchymal stromal cells (eAD-MSC) cultured with fetal bovine serum (FBS), medium-concentration platelet lysate (MPL) and high concentration platelet lysate (HPL). Microscopic images (10×) of confluent cells for FBS **(A)**, MPL **(B)** and HPL **(C)** conditions. Macroscopic images of cell colonies cultured with FBS **(D)**, MPL **(E)** and HPL **(F)**.

## References

[1] Voga M Adamic N Vengust M Majdic G. Stem cells in veterinary medicine—current state and treatment options. Front Vet Sci. (2020) 7:278. doi: 10.3389/fvets.2020.00278, PMID: 32656249 PMC7326035

[2] Cequier A Sanz C Rodellar C Barrachina L. The usefulness of mesenchymal stem cells beyond the musculoskeletal system in horses. Animals. (2021) 11:931. doi: 10.3390/ani11040931, PMID: 33805967 PMC8064371

[3] Cassano JM Schnabel LV Goodale MB Fortier LA. The immunomodulatory function of equine MSCs is enhanced by priming through an inflammatory microenvironment or TLR3 ligand. Vet Immunol Immunopathol. (2018) 195:33–9. doi: 10.1016/j.vetimm.2017.10.003 29249315

[4] Arzi B Peralta S Fiani N Vapniarsky N Taechangam N Delatorre U. A multicenter experience using adipose-derived mesenchymal stem cell therapy for cats with chronic, non-responsive gingivostomatitis. Stem Cell Res Ther. (2020) 11:115. doi: 10.1186/s13287-020-01623-9, PMID: 32169089 PMC7071622

[5] Barrachina L Remacha AR Romero A Vitoria A Albareda J Prades M. Assessment of effectiveness and safety of repeat administration of proinflammatory primed allogeneic mesenchymal stem cells in an equine model of chemically induced osteoarthritis. BMC Vet Res. (2018) 14:241. doi: 10.1186/s12917-018-1556-3, PMID: 30119668 PMC6098603

[6] Hemeda H Giebel B Wagner W. Evaluation of human platelet lysate versus fetal bovine serum for culture of mesenchymal stromal cells. Cytotherapy. (2014) 16:170–80. doi: 10.1016/j.jcyt.2013.11.004, PMID: 24438898

[7] Pilgrim CR McCahill KA Rops JG Dufour JM Russell KA Koch TG. A review of fetal bovine serum in the culture of mesenchymal stromal cells and potential alternatives for veterinary medicine. Front Vet Sci. (2022) 9:859025. doi: 10.3389/fvets.2022.859025, PMID: 35591873 PMC9111178

[8] Jochems CEA van der Valk JB Stafleu FR Baumans V. The use of fetal bovine serum: ethical or scientific problem? Altern Lab Anim. (2002) 30:219–27. doi: 10.1177/026119290203000208 11971757

[9] Iudicone P Fioravanti D Bonanno G Miceli M Lavorino C Totta P. Pathogen-free, plasma-poor platelet lysate and expansion of human mesenchymal stem cells. J Transl Med. (2014) 12:28. doi: 10.1186/1479-5876-12-28, PMID: 24467837 PMC3918216

[10] Hagen A Lehmann H Aurich S Bauer N Melzer M Moellerberndt J. Scalable production of equine platelet lysate for multipotent mesenchymal stromal cell culture. Front Bioeng Biotechnol. (2021) 8:613621. doi: 10.3389/fbioe.2020.613621, PMID: 33553119 PMC7859354

[11] Even KM Gaesser AM Ciamillo SA Linardi RL Ortved KF. Comparing the immunomodulatory properties of equine BM-MSCs culture expanded in autologous platelet lysate, pooled platelet lysate, equine serum and fetal bovine serum supplemented culture media. Front Vet Sci. (2022) 9:958724. doi: 10.3389/fvets.2022.958724, PMID: 36090170 PMC9453159

[12] Russell KA Koch TG. Equine platelet lysate as an alternative to fetal bovine serum in equine mesenchymal stromal cell culture - too much of a good thing? Equine Vet J. (2016) 48:261–4. doi: 10.1111/evj.12440, PMID: 25772755

[13] Moellerberndt J Hagen A Niebert S Büttner K Burk J. Cytokines in equine platelet lysate and related blood products. Front Vet Sci. (2023) 10:1117829. doi: 10.3389/fvets.2023.1117829, PMID: 36968472 PMC10033973

[14] Moellerberndt J Niebert S Fey K Hagen A Burk J. Impact of platelet lysate on immunoregulatory characteristics of equine mesenchymal stromal cells. Front Vet Sci. (2024) 11:1385395. doi: 10.3389/fvets.2024.1385395 38725585 PMC11079816

[15] Seo JP Tsuzuki N Haneda S Yamada K Furuoka H Tabata Y. Comparison of allogeneic platelet lysate and fetal bovine serum for in vitro expansion of equine bone marrow-derived mesenchymal stem cells. Res Vet Sci. (2013) 95:693–8. doi: 10.1016/j.rvsc.2013.04.024 23683731

[16] Naskou MC Sumner SM Chocallo A Kemelmakher H Thoresen M Copland I. Platelet lysate as a novel serum-free media supplement for the culture of equine bone marrow-derived mesenchymal stem cells. Stem Cell Res Ther. (2018) 9:75. doi: 10.1186/s13287-018-0823-3, PMID: 29566772 PMC5863827

[17] Sumner SM Naskou MC Thoresen M Copland I Peroni JF. Platelet lysate obtained via plateletpheresis performed in standing and awake equine donors. Transfusion. (2017) 57:1755–62. doi: 10.1111/trf.14124, PMID: 28439897

[18] Doucet C Ernou I Zhang Y Llense JR Begot L Holy X. Platelet lysates promote mesenchymal stem cell expansion: a safety substitute for animal serum in cell-based therapy applications. J Cell Physiol. (2005) 205:228–36. doi: 10.1002/jcp.20391, PMID: 15887229

[19] Yaneselli K Barrachina L Remacha AR Algorta A Vitoria A Cequier A. Effect of allogeneic platelet lysate on equine bone marrow derived mesenchymal stem cell characteristics, including immunogenic and immunomodulatory gene expression profile. Vet Immunol Immunopathol. (2019) 217:109944. doi: 10.1016/j.vetimm.2019.109944, PMID: 31563725

[20] Hagen A Holland H Brandt VP Doll CU Häußler TC Melzer M. Platelet lysate for mesenchymal stromal cell culture in the canine and equine species: analogous but not the same. Animals. (2022) 12:189. doi: 10.3390/ani12020189, PMID: 35049811 PMC8773277

[21] Hemeda H Kalz J Walenda G Lohmann M Wagner W. Heparin concentration is critical for cell culture with human platelet lysate. Cytotherapy. (2013) 15:1174–81. doi: 10.1016/j.jcyt.2013.05.006, PMID: 23845186

[22] Ranera B Ordovás L Lyahyai J Bernal ML Fernandes F Remacha AR. Comparative study of equine bone marrow and adipose tissue-derived mesenchymal stromal cells. Equine Vet J. (2012) 44:33–42. doi: 10.1111/j.2042-3306.2010.00353.x 21668489

[23] Vidal MA Kilroy GE Johnson JR Lopez MJ Moore RM Gimble JM. Cell growth characteristics and differentiation frequency of adherent equine bone marrow-derived mesenchymal stromal cells: adipogenic and osteogenic capacity. Vet Surg. (2006) 35:601–10. doi: 10.1111/j.1532-950X.2006.00197.x, PMID: 17026544

[24] Guercio A Di BS Casella S Di MP Russo C Piccione G. Canine mesenchymal stemcells (MSCS): characterization in relation to donor age and adipose tissue-harvesting site. Cell Biol Int. (2013) 37:789–98. doi: 10.1002/cbin.10090, PMID: 23505013

[25] Algorta A Artigas R Rial A Brandl S Rodellar C Benavides U. Isolation and characterization of feline dental pulp stem cells. J Feline Med Surg. (2023) 25:1098612X221150625. doi: 10.1177/1098612X221150625, PMID: 36745130 PMC10812064

[26] Yaneselli KM Kuhl CP Terraciano PB de Oliveira FS Pizzato SB Pazza K. Comparison of the characteristics of canine adipose tissue-derived mesenchymal stem cells extracted from different sites and at different passage numbers. J Vet Sci. (2018) 19:13–20. doi: 10.4142/jvs.2018.19.1.13, PMID: 28693305 PMC5799390

[27] Remacha AR Barrachina L Álvarez-Arguedas S Ranera B Romero A Vázquez FJ. Expression of genes involved in immune response and *in vitro* immunosuppressive effect of equine MSCs. Vet Immunol Immunopathol. (2015) 165:107–18. doi: 10.1016/j.vetimm.2015.04.004, PMID: 25977164

[28] Barrachina L Remacha AR Romero A Vázquez FJ Albareda J Prades M. Effect of inflammatory environment on equine bone marrow derived mesenchymal stem cells immunogenicity and immunomodulatory properties. Vet Immunol Immunopathol. (2016) 171:57–65. doi: 10.1016/j.vetimm.2016.02.007, PMID: 26964718

[29] Livak KJ Schmittgen TD. Analysis of relative gene expression data using real-time quantitative PCR and the 2-ΔΔCT method. Methods. (2001) 25:402–8. doi: 10.1006/meth.2001.1262 11846609

[30] Del Bue M Riccò S Conti V Merli E Ramoni R Grolli S. Platelet lysate promotes in vitro proliferation of equine mesenchymal stem cells and tenocytes. Vet Res Commun. (2007) 31 Suppl 1:289–92. doi: 10.1007/s11259-007-0099-z, PMID: 17682897

[31] Gilbertie JM Long JM Schubert AG Berglund AK Schaer TP Schnabel LV. Pooled platelet-rich plasma lysate therapy increases synoviocyte proliferation and hyaluronic acid production while protecting chondrocytes from synoviocyte-derived inflammatory mediators. Front Vet Sci. (2018) 5:150. doi: 10.3389/fvets.2018.00150, PMID: 30023361 PMC6039577

[32] Giraldo CE López C Álvarez ME Samudio IJ Prades M Carmona JU. Effects of the breed, sex and age on cellular content and growth factor release from equine pure-platelet rich plasma and pure-platelet rich gel. BMC Vet Res. (2013) 9:29. doi: 10.1186/1746-6148-9-29, PMID: 23402541 PMC3577464

[33] Bourin P Bunnell BA Casteilla L Dominici M Katz AJ March KL. Stromal cells from the adipose tissue-derived stromal vascular fraction and culture expanded adipose tissue-derived stromal/stem cells: a joint statement of the International Federation for Adipose Therapeutics and Science (IFATS) and the International Society for Cellular Therapy (ISCT). Cytotherapy. (2013) 15:641–8. doi: 10.1016/j.jcyt.2013.02.006, PMID: 23570660 PMC3979435

[34] Dominici M Le Blanc K Mueller I Slaper-Cortenbach I Marini FC Krause DS. Minimal criteria for defining multipotent mesenchymal stromal cells. Int Soc Cell Ther Posit Stat Cytother. (2006) 8:315–7. doi: 10.1080/14653240600855905, PMID: 16923606

[35] Cequier A Vázquez FJ Romero A Vitoria A Bernad E García-Martínez M. The immunomodulation–immunogenicity balance of equine mesenchymal stem cells (MSCs) is differentially affected by the immune cell response depending on inflammatory licensing and major histocompatibility complex (MHC) compatibility. Front Vet Sci. (2022) 9:957153. doi: 10.3389/fvets.2022.957153, PMID: 36337202 PMC9632425

[36] Kamm JL Parlane NA Riley CB Gee EK Dittmer KE McIlwraith CW. Blood type and breed-associated differences in cell marker expression on equine bone marrow-derived mesenchymal stem cells including major histocompatibility complex class II antigen expression. PLoS One. (2019) 14:e0225161. doi: 10.1371/journal.pone.0225161, PMID: 31747418 PMC6867698

[37] Schnabel LV Pezzanite LM Antczak DF Felippe MJB Fortier LA. Equine bone marrow-derived mesenchymal stromal cells are heterogeneous in MHC class II expression and capable of inciting an immune response in vitro. Stem Cell Res Ther. (2014) 5:13. doi: 10.1186/scrt402, PMID: 24461709 PMC4055004

[38] Barrachina L Cequier A Romero A Vitoria A Zaragoza P Vázquez FJ. Allo-antibody production after intraarticular administration of mesenchymal stem cells (MSCs) in an equine osteoarthritis model: effect of repeated administration, MSC inflammatory stimulation, and equine leukocyte antigen (ELA) compatibility. Stem Cell Res Ther. (2020) 11:52. doi: 10.1186/s13287-020-1571-8, PMID: 32028995 PMC7006079

[39] Cassano JM Fortier LA Hicks RB Harman RM Van de Walle GR. Equine mesenchymal stromal cells from different tissue sources display comparable immune-related gene expression profiles in response to interferon gamma (IFN)-γ. Vet Immunol Immunopathol. (2018) 202:25–30. doi: 10.1016/j.vetimm.2018.06.008, PMID: 30078595

[40] Caffi V Espinosa G Gajardo G Morales N Durán MC Uberti B. Pre-conditioning of equine bone marrow-derived mesenchymal stromal cells increases their immunomodulatory capacity. Front Vet Sci. (2020) 7:318. doi: 10.3389/fvets.2020.00318, PMID: 32656251 PMC7325884

[41] Jammes M Contentin R Audigié F Cassé F Galéra P. Effect of pro-inflammatory cytokine priming and storage temperature of the mesenchymal stromal cell (MSC) secretome on equine articular chondrocytes. Front Bioeng Biotechnol. (2023) 11:1204737. doi: 10.3389/fbioe.2023.1204737, PMID: 37720315 PMC10502223

[42] Berglund AK Schnabel LV. Allogeneic major histocompatibility complex-mismatched equine bone marrow-derived mesenchymal stem cells are targeted for death by cytotoxic anti-major histocompatibility complex antibodies. Equine Vet J. (2017) 49:539–44. doi: 10.1111/evj.12647, PMID: 27862236 PMC5425313

