# Supplementary figures and images for: Impact of different formulations of platelet lysate on proliferative and immune profile of equine mesenchymal stromal cells
Source: Front Vet Sci. 2024 Aug 5;11:1410855. doi: 10.3389/fvets.2024.1410855 (PMC11330840; doi:10.3389/fvets.2024.1410855)

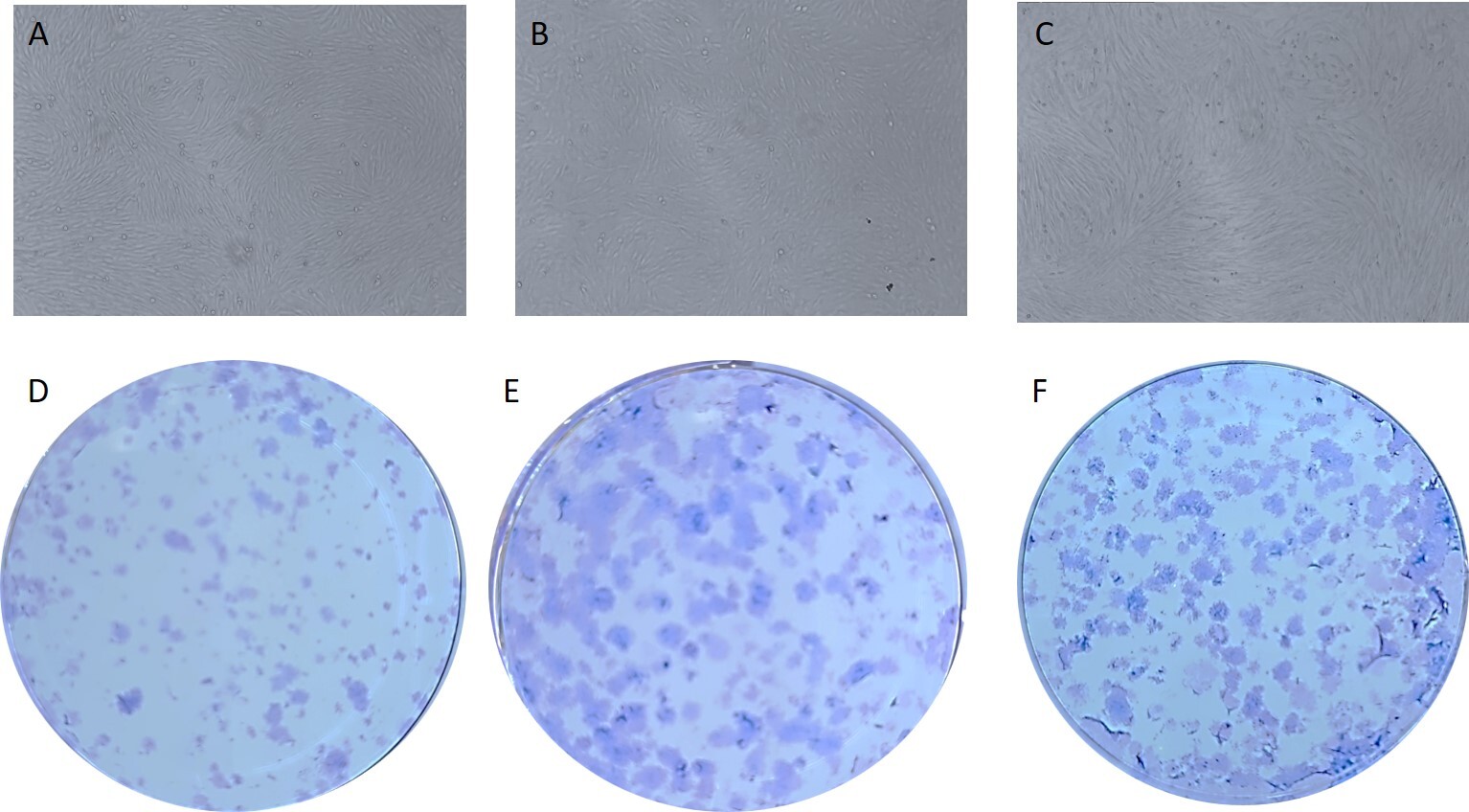

Supplement: Supplementary FIGURE S1 — Equine adipose tissue-derived mesenchymal stromal cells (eAD-MSC) cultured with fetal bovine serum (FBS), medium-concentration platelet lysate (MPL) and high concentration platelet lysate (HPL). Microscopic images (10×) of confluent cells for FBS (A), MPL (B) and HPL (C) conditions. Macroscopic images of cell colonies cultured with FBS (D), MPL (E) and HPL (F). [file Image_1.JPEG]
