# Supplementary material for: Impact of different formulations of platelet lysate on proliferative and immune profile of equine mesenchymal stromal cells
Source: Front Vet Sci. 2024 Aug 5;11:1410855. doi: 10.3389/fvets.2024.1410855 (PMC11330840; doi:10.3389/fvets.2024.1410855)
Supplement: Supplementary file 2 [file Table_1.DOCX]

Supplementary Material

**Impact of different formulations of platelet lysate on proliferative and immune profile of equine mesenchymal stromal cells**

Kevin Yaneselli*, Gimena Ávila1, Andrea Rossi, Analía Rial, Sabrina Castro, María José Estradé, Gonzalo Suárez, Agustina Algorta

*Correspondence: [kevin.yaneselli@fvet.edu.uy](mailto:kevin.yaneselli@fvet.edu.uy)

**RESULTS 3.1**

**Table I:** Estimators of Incidence Rate Ratios and 95% confidence interval (CI) platelet concentrations obtained from the parameters of statistical models.

Reference: Whole blood

|  | **PLT (10^3)** | | |
| --- | --- | --- | --- |
| *Predictors* | *Incidence Rate Ratios* | *CI* | *p* |
| TREAT_HPL | 7.84 ^***^ | 7.34 – 8.38 | **<0.001** |
| TREAT_MPL | 3.99 ^***^ | 3.72 – 4.28 | **<0.001** |
| TREAT_Plasma | 1.23 ^***^ | 1.13 – 1.34 | **<0.001** |
| **Random Effects** | | | |
| σ^2^ | 0.00 | | |
| τ_00_ _ID_ | 0.06 | | |
| ICC | 1.00 | | |
| N _ID_ | 5 | | |
| Observations | 20 | | |
| Marginal R^2^ / Conditional R^2^ | 0.924 / 1.000 | | |
| ** p<0.05   ** p<0.01   *** p<0.001* | | | |

**Table II:** Estimators of Incidence Rate Ratios and 95% confidence interval (CI) WBC concentrations obtained from the parameters of statistical models.

Reference: Whole blood

|  | **WBC** | | |
| --- | --- | --- | --- |
| *Predictors* | *Incidence Rate Ratios* | *CI* | *p* |
| Whole blood | *Reference* |  |  |
| HPL | 1.56 ^***^ | 1.54 – 1.58 | **<0.001** |
| MPL | 0.80 ^***^ | 0.79 – 0.82 | **<0.001** |
| Plasma | 0.17 ^***^ | 0.16 – 0.17 | **<0.001** |
| **Random Effects** | | | |
| σ^2^ | 0.00 | | |
| τ_00_ _ID_ | 0.09 | | |
| ICC | 1.00 | | |
| N _ID_ | 5 | | |
| Observations | 20 | | |
| Marginal R^2^ / Conditional R^2^ | 0.886 / 1.000 | | |
| ** p<0.05   ** p<0.01   *** p<0.001* | | | |

**Table III:** Estimators and 95% confidence interval (CI) TGF-β concentrations obtained from the parameters of statistical models.

Reference: Plasma

|  | **TGF-β** | | |
| --- | --- | --- | --- |
| *Predictors* | *Estimates* | *CI* | *p* |
| Plasma | *Reference* |  |  |
| HPC | 16896.60 ^*^ | 1531.16 – 32262.04 | **0.033** |
| HPL | 13026.60 | -2338.84 – 28392.04 | 0.092 |
| MPC | 770.00 | -14595.44 – 16135.44 | 0.917 |
| MPL | 5311.60 | -10053.84 – 20677.04 | 0.477 |
| **Random Effects** | | | |
| σ^2^ | 133724264.82 | | |
| τ_00_ _ID_ | 0.00 | | |
| N _ID_ | 5 | | |
| Observations | 25 | | |
| Marginal R^2^ / Conditional R^2^ | 0.259 / NA | | |
| ** p<0.05   ** p<0.01   *** p<0.001* | | | |

**Table IV:** Estimators and 95% confidence interval (CI) PDGF concentrations obtained from the parameters of statistical models.

Reference: Plasma

|  | **PDGF** | | |
| --- | --- | --- | --- |
| *Predictors* | *Estimates* | *CI* | *p* |
| Plasma | *Reference* |  |  |
| HPC | 1995.40 ^***^ | 1173.56 – 2817.24 | **<0.001** |
| HPL | 850.60 ^*^ | 28.76 – 1672.44 | **0.043** |
| MPC | 1189.00 ^**^ | 367.16 – 2010.84 | **0.007** |
| MPL | 379.00 | -442.84 – 1200.84 | 0.345 |
| **Random Effects** | | | |
| σ^2^ | 382551.10 | | |
| τ_00_ _ID_ | 62220.72 | | |
| ICC | 0.14 | | |
| N _ID_ | 5 | | |
| Observations | 25 | | |
| Marginal R^2^ / Conditional R^2^ | 0.526 / 0.592 | | |
| ** p<0.05   ** p<0.01   *** p<0.001* | | | |

**RESULTS 3.2**

**Table V:** Estimators of Incidence Rate Ratios and 95% confidence interval (CI) of cell proliferation (MTT) obtained from the parameters of statistical models.

Reference: PASSAGE3 and FBS

|  | **MTT** | | |
| --- | --- | --- | --- |
| *Predictors* | *Incidence Rate Ratios* | *CI* | *p* |
| PASSAGE4 | 0.90 ^***^ | 0.89 – 0.90 | **<0.001** |
| FBS | *Reference* |  |  |
| HPL | 0.74 ^***^ | 0.74 – 0.74 | **<0.001** |
| TREAT_HPL:PASSAGE4 | 1.13 ^***^ | 1.13 – 1.14 | **<0.001** |
| MPL | 0.59 ^***^ | 0.59 – 0.59 | **<0.001** |
| TREAT_MPL:PASSAGE4 | 1.04 ^***^ | 1.04 – 1.05 | **<0.001** |
| **Random Effects** | | | |
| σ^2^ | 0.00 | | |
| τ_00_ _ID_ | 0.19 | | |
| τ_11_ _ID.DAYS_ | 0.00 | | |
| ρ_01_ _ID_ | -0.23 | | |
| ICC | 1.00 | | |
| N _ID_ | 7 | | |
| Observations | 294 | | |
| Marginal R^2^ / Conditional R^2^ | 0.190 / 1.000 | | |
| ** p<0.05   ** p<0.01   *** p<0.001* | | | |

**Table VI:** Estimates and 95% confidence interval (CI) of cell proliferation (DT) obtained from the parameters of statistical models.

Reference: PASSAGE2 and FBS

|  | **DT** | | |
| --- | --- | --- | --- |
| *Predictors* | *Estimates* | *CI* | *p* |
| PASSAGE3 | -0.75 ^**^ | -1.20 – -0.29 | **0.002** |
| PASSAGE4 | -0.80 ^***^ | -1.26 – -0.34 | **0.001** |
| FBS | *Reference* |  |  |
| HPL | 0.01 | -0.45 – 0.47 | 0.965 |
| TREAT HPL:PASSAGE3 | 0.13 | -0.51 – 0.78 | 0.678 |
| TREAT HPL:PASSAGE4 | -0.06 | -0.70 – 0.59 | 0.863 |
| MPL | 0.16 | -0.30 – 0.62 | 0.489 |
| TREAT MPL:PASSAGE3 | 0.31 | -0.34 – 0.95 | 0.347 |
| TREAT MPL:PASSAGE4 | 0.08 | -0.56 – 0.73 | 0.801 |
| **Random Effects** | | | |
| σ^2^ | 0.18 | | |
| τ_00_ _ID_ | 0.03 | | |
| ICC | 0.14 | | |
| N _ID_ | 7 | | |
| Observations | 63 | | |
| Marginal R^2^ / Conditional R^2^ | 0.391 / 0.478 | | |
| ** p<0.05   ** p<0.01   *** p<0.001* | | | |

**Table VII:** Estimators of Incidence Rate Ratios and 95% confidence interval (CI) of cell proliferation (CFU-F) obtained from the parameters of statistical models.

Reference: FBS

|  | **CFU-F** | | |
| --- | --- | --- | --- |
| *Predictors* | *Incidence Rate Ratios* | *CI* | *p* |
| FBS | *Reference* |  |  |
| HPL | 1.16 | 0.98 – 1.36 | 0.078 |
| MPL | 1.17 | 1.00 – 1.37 | 0.055 |
| **Random Effects** | | | |
| σ^2^ | 0.02 | | |
| τ_00_ _ID_ | 0.01 | | |
| ICC | 0.32 | | |
| N _ID_ | 6 | | |
| Observations | 18 | | |
| Marginal R^2^ / Conditional R^2^ | 0.159 / 0.426 | | |
| ** p<0.05   ** p<0.01   *** p<0.001* | | | |

**RESULTS 3.4**

**3.4.3 Immunomodulatory cytokine quantification**

**Table VIII:** Estimates and 95% confidence interval (CI) IL-6 concentrations obtained from the parameters of statistical models.

Reference: PASSAGE3 and FBS

|  | **IL-6** | | |
| --- | --- | --- | --- |
| *Predictors* | *Estimates* | *CI* | *p* |
| PASSAGE4 | -5.80 | -73.03 – 61.43 | 0.861 |
| FBS | *Reference* |  |  |
| HPL | 214.57 ^***^ | 147.34 – 281.80 | **<0.001** |
| TREATHPL:PASSAGE4 | -79.63 | -174.71 – 15.45 | 0.097 |
| MPL | 136.76 ^**^ | 57.06 – 216.46 | **0.002** |
| TREATMPL:PASSAGE4 | -108.20 | -219.69 – 3.29 | 0.057 |
| **Random Effects** | | | |
| σ^2^ | 3770.31 | | |
| τ_00_ _ID_ | 449.74 | | |
| ICC | 0.11 | | |
| N _ID_ | 7 | | |
| Observations | 36 | | |
| Marginal R^2^ / Conditional R^2^ | 0.643 / 0.681 | | |
| ** p<0.05   ** p<0.01   *** p<0.001* | | | |

**Table IX:** Estimates and 95% confidence interval (CI) IL-10 concentrations obtained from the parameters of statistical models.

Reference: PASSAGE3 and FBS

|  | **IL-10** | | |
| --- | --- | --- | --- |
| *Predictors* | *Estimates* | *CI* | *p* |
| PASSAGE4 | -0.14 | -0.86 – 0.57 | 0.685 |
| FBS | *Reference* |  |  |
| HPL | 3.43 ^***^ | 2.71 – 4.14 | **<0.001** |
| TREATHPL:PASSAGE4 | 0.29 | -0.72 – 1.30 | 0.567 |
| MPL | 0.08 | -0.77 – 0.93 | 0.850 |
| TREATMPL:PASSAGE4 | -0.11 | -1.29 – 1.08 | 0.854 |
| **Random Effects** | | | |
| σ^2^ | 0.43 | | |
| τ_00_ _ID_ | 0.13 | | |
| ICC | 0.23 | | |
| N _ID_ | 7 | | |
| Observations | 36 | | |
| Marginal R^2^ / Conditional R^2^ | 0.849 / 0.884 | | |
| ** p<0.05   ** p<0.01   *** p<0.001* | | | |

**Table X:** Estimates and 95% confidence interval (CI) TNF-α concentrations obtained from the parameters of statistical models.

Reference: PASSAGE3 and FBS

|  | ***TNF-α*** | | |
| --- | --- | --- | --- |
| *Predictors* | *Estimates* | *CI* | *p* |
| PASSAGE4 | -5.43 | -26.14 – 15.28 | 0.595 |
| FBS | *Reference* |  |  |
| HPL | 101.71 ^***^ | 81.01 – 122.42 | **<0.001** |
| TREAT HPL:PASSAGE4 | 33.14 ^*^ | 3.86 – 62.43 | **0.028** |
| MPL | -2.02 | -26.31 – 22.28 | 0.866 |
| TREAT MPL:PASSAGE4 | 2.43 | -31.91 – 36.77 | 0.886 |
| **Random Effects** | | | |
| σ^2^ | 357.65 | | |
| τ_00_ _ID_ | 1.33 | | |
| ICC | 0.00 | | |
| N _ID_ | 7 | | |
| Observations | 36 | | |
| Marginal R^2^ / Conditional R^2^ | 0.907 / 0.908 | | |
| ** p<0.05   ** p<0.01   *** p<0.001* | | | |
